# Targeting gut microbiota: new therapeutic opportunities in multiple sclerosis

**DOI:** 10.1080/19490976.2023.2274126

**Published:** 2023-11-18

**Authors:** Dorota Kujawa, Lukasz Laczmanski, Slawomir Budrewicz, Anna Pokryszko-Dragan, Maria Podbielska

**Affiliations:** a Laboratory of Genomics & Bioinformatics, Ludwik Hirszfeld Institute of Immunology & Experimental Therapy, Polish Academy of Sciences, Wroclaw, Poland; b Department of Neurology, Wroclaw Medical University, Wroclaw, Poland; c Laboratory of Microbiome Immunobiology, Ludwik Hirszfeld Institute of Immunology & Experimental Therapy, Polish Academy of Sciences, Wroclaw, Poland

**Keywords:** Antibiotics, diet, disease-modifying treatment, fecal microbiota transplantation, gut microbiome, microbiota, multiple sclerosis, prebiotics, probiotics

## Abstract

Multiple sclerosis (MS) causes long-lasting, multifocal damage to the central nervous system. The complex background of MS is associated with autoimmune inflammation and neurodegeneration processes, and is potentially affected by many contributing factors, including altered composition and function of the gut microbiota. In this review, current experimental and clinical evidence is presented for the characteristics of gut dysbiosis found in MS, as well as for its relevant links with the course of the disease and the dysregulated immune response and metabolic pathways involved in MS pathology. Furthermore, therapeutic implications of these investigations are discussed, with a range of pharmacological, dietary and other interventions targeted at the gut microbiome and thus intended to have beneficial effects on the course of MS.

## Introduction

Multiple sclerosis (MS) is one of the most common chronic diseases of the central nervous system (CNS). Disseminated lesions throughout the brain and spinal cord, comprising inflammatory demyelination and axonal loss, cause a range of symptoms of neurological deficit and result in accumulating disability.^1^ The course of the disease is long-lasting and highly variable, most frequently relapsing-remitting but at some stages also progressive. The core process underlying MS pathology is associated with the dysregulated, autoreactive response of the immune system, targeted at the CNS antigens. A slowly expanding neurodegenerative process accompanies immune-mediated inflammation. However, the etiology of the disease appears to be complex, and some of its aspects still need to be fully elucidated, despite substantial progress in this field.^2^

In recent years, there has been a consensus about the importance of gene–environment interactions in triggering and modulating the autoimmune response, relevant to the development of MS. Genetic factors are supposed to account for ca. 30% of MS risk. So far, up to 200 genetic variants (mainly associated with the immune system, e.g. *HLA-DR* and *DQ* alleles) and their epigenetic modifications have been identified as associated with the risk of MS or modulation of its course. There is also growing evidence of the significant contributing role of environmental factors, linked with genetic predisposition, in determining the onset and progression of MS. These factors include: smoking, exposure to sunlight, level of vitamin D3, stress, obesity, diet components and infections.^2–4^

According to the hygiene hypothesis, infections in childhood and adolescence enhance later regulatory properties of the immune system; thus, limited exposure to infectious pathogens may facilitate autoimmune diseases.^4^ Moreover, several mechanisms are suggested as the link between infections and MS onset or its further exacerbations. These include “molecular mimicry”, epitope spread, “bystander activation”, superantigen properties of pathogens, as well as expression of cryptic antigens due to injury of tissues. All these mechanisms may contribute to the activation of autoreactive immunocompetent cells, their proliferation and migration through the blood-brain barrier (BBB), resulting in inflammatory demyelination in the CNS.^3^ Research focused on viral agents has shown that Epstein-Barr virus (EBV) infection/seroconversion is a main driver for the risk of MS, exposure to HHV6 and HSV-1 moderately increases this risk, while prior infection with CMV seems to have a protective effect.^2,3,5^ Bacterial agents are suggested to have an impact as potential risk factors for MS mainly through their toxins. In animal models, toxins from *Staphylococcus* and *Clostridium perfringens* were reported to stimulate autoreactive T cells, cross the BBB and bind to myelinated neuronal fibers. Conversely, pertussis toxin exerted a protective effect by reducing T cell infiltration, activating microglia and upregulating regulatory cells.^6^ *Helicobacter pylori*, which occurs with lower prevalence in MS subjects than in the general population, was also shown to ameliorate experimental inflammatory demyelination.^3^

Recently, increasing attention has been paid to the role of microorganisms living in the human intestine (the gut microbiota) as well as their genomes, metabolites and the surrounding conditions (defined as the gut microbiome)^7^ in the background of the CNS diseases.

The gut-brain axis comprises bidirectional communication between the gastrointestinal system and the CNS, including endocrine, metabolic, immune, and neurotransmitters links. The gut microbiome plays a significant role in these interactions, e.g. through vagal stimulation, the release of metabolites into the circulation and stimulating immunocompetent cells within the intestinal wall. There is evidence of the impact of the gut microbiome on the maturation and differentiation of neurons and glial cells, functional integrity of the BBB, as well as maintaining the balance between pro- and anti-inflammatory components of the immune response. Thus, the gut microbiome’s relevance in MS development and dynamics seems particularly appealing.^8,9^

**Figure 1.**
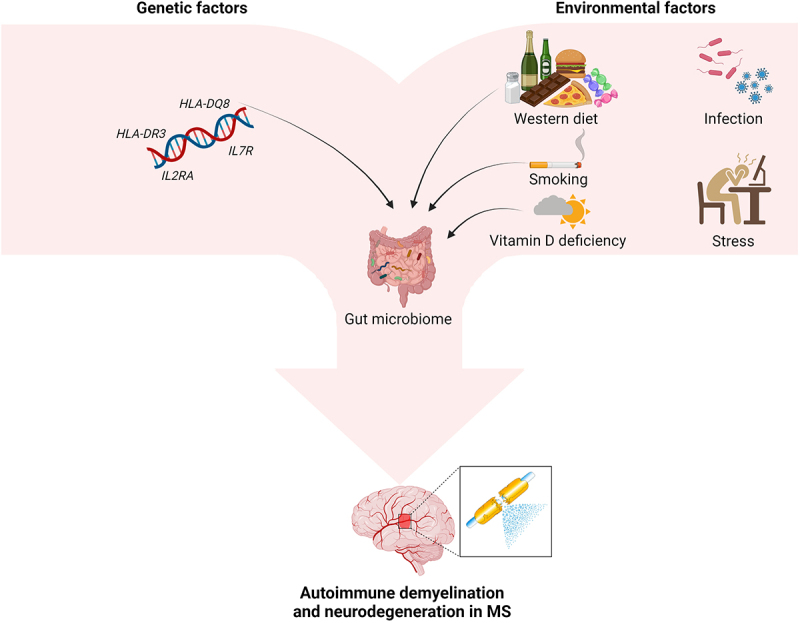
Contribution of genetic and environmental factors to development of MS.

Some interesting links have been observed between the gut microbiome and genetic or environmental risk factors for MS (Figure 1). Overall, complex interactions between host and gut microbiota affect composition and function of the latter. Metabolism of bacteria is based on the substrates determined by the host genetics, while host or bacterial gene expression may be mutually regulated by specific miRNAs.^10^ The role of these interactions in determining susceptibility to CNS autoimmunity was demonstrated in animal models. Evidence was provided for a significant role of the *HLA-DQ8* and *HLA-DR3* genes in shaping murine gut microbiota as well as their linked contribution to EAE susceptibility or resistance.^11^ Furthermore, diverse microbial profiles associated with specific genotypes were identified in mice, and the introduction of incompatible bacterial strains (e.g. *Lactobacillus reuteri*) in a genetically susceptible host was demonstrated to contribute to EAE exacerbation.^12^

Genetic and environmental factors, and their mutual interactions, are considered to play a significant contributing role in initiation and development of MS. The evidence of links between some of these factors and the gut microbiome supports the hypothesis of its relevance for the processes underlying MS background.

Genome-wide association studies revealed some associations between microbiota composition and variants of the vitamin D receptor (*VDR*) gene.^13^ Moreover, the level of vitamin D3 affects intestinal calcium absorption and thus intestinal motility, as well as the integrity of the mucosal intestinal barrier. Thus, vitamin D3 deficiency contributes to gut stasis with a rise in intestinal permeability for bacteria and their products. Smoking, considered as another risk factor for MS (due to the impact on pro-inflammatory cytokines and migration of T cells through the BBB), may modify the content and function of gut microbiota through toxic substances contained in tobacco smoke and/or immune-mediated mechanisms.^2,4,14^

It is still a matter of debate whether the gut microbiome acts as a triggering and modulating factor for the autoimmune response involved in the background of MS, or whether perhaps the gut dysbiosis develops as a consequence of the disturbed balance between pro- and anti-inflammatory mechanisms and metabolic alterations in the course of disease. However, investigation of the composition and functions of the gut microbiome in people with MS (pwMS) may provide a better insight into the background of MS and dynamics of its clinical course.^8,15^

Despite recent progress in MS management, there are still some challenges ahead, associated inter alia with individual differences in the course of disease and response to treatment. There is an ongoing search for biomarkers for the disease activity and progression. In the current therapeutic guidelines for MS, a trend for personalized and complex treatment strategies is highlighted, with disease-modifying therapies (DMT) complemented with modification of lifestyle.^16,17^ In view of that, the gut microbiota profile could be considered as a signature of individual immune or metabolic properties, and thus a promising marker for choice of DMT or supportive therapeutic interventions.

Therefore, in this review, we discuss the current evidence on the role of the gut microbiome in the processes underlying MS pathology, focusing on emerging potential therapeutic implications.

## Methods

The online literature search was conducted using PubMed and Scopus databases, covering the publication period from the start of 2010 until 1st March 2023. The following combination of key search terms was applied: “multiple sclerosis”, “gut microbiome/microbiota” and “treatment”. Having excluded conference abstracts and papers written in languages other than English, the reviews and original research studies were screened and analyzed for their relevance to the topic. In addition, the reference lists from the retrieved publications were investigated and further potentially relevant papers were identified. Initially the literature search was performed by the first author, with its results reviewed and further stages of the search verified by the other authors.

## Characteristics of the gut microbiome in MS

### Composition of the gut microbiota in MS patients

Since 2015 the composition of the gut microbiota in MS has become a subject of considerable interest. One of the earliest studies indicated that epsilon toxin produced by *Clostridium perfringens* (a gut commensal) can modulate the integrity of the BBB, reach the CNS and act as a potential trigger of the MS-related autoimmune response.^18^ Several subsequent reports showed that the composition of the main bacterial taxa in the intestine was significantly altered in MS patients compared to healthy subjects (HS) (Table 1). The identified changes were heterogenous and differed depending on the studied population and methodology (inter alia, sequencing platform and investigated 16S rRNA gene region). Despite this diversity, a consensus seems to be emerging at the taxonomic level concerning the relative abundance of particular bacterial strains. The most frequently identified depleted bacterial taxa included *Bacteroides*,^19–25^ *Faecalibacterium*,^21,22,25–27^ *Prevotella*^23,24,28,29^ and *Roseburia*,^21,22,26,30^ whereas the enriched ones included *Akkermansia*^19,28,30–34^ and *Streptococcus*.^23,24,30,33,35,36^ The composition of gut microbiota differed between the patients with various types of MS.^30,32,33,39^ Relapsing-remitting type (RRMS) was associated with a decreased amount of *Faecalibacterium prausnitzii*, *Eubacterium rectale* and *Roseburia*,^21,32^ while primary progressive type (PPMS) was associated with an elevated level of *Enterobacteriaceae* and *Clostridium g24 FCEY* and a decreased level of *Blautia* and *Agathobaculum*.^32^ In those with secondary progressive (SPMS) type, a relative increase of *Clostridium and Streptococcus* strains was observed.^33^

**Table 1.** Alterations of the gut microbiota in MS patients without specified treatment.

Altered bacteria	Identified in / Compared to	Reference
*Clostridium perfringens type B***↑**	26 RRMS and 4 SPMS/31 HS	^18^
*Akkermansia muciniphila***↑**, *Acinetobacter calcoaceticus***↑**,*Parabacteroides***↓**	71 RRMS/71 HS	^19^
*Atopobium***↑**, *Bifidobacterium***↑**, *Bacteroidaceae***↓**	54 MS/HS (number of control not provided)	^20^
*Bacteroides fragilis***↓**, *Butyrivibrio***↓**, *Clostridium***↓**, *Coprococcus***↓**, *Eubacterium rectale***↓**,*Faecalibacterium prausnitzii***↓**, *Lawsonella***↑**, *Roseburia***↓**	129 RRMS/58 HS	^21^
*Bacteroides***↓**, *Faecalibacterium prausnitzii***↓**, *Roseburia***↓**	25 RRMS/14 HS	^22^
*Firmicutes/Bacteroides ratio***↑**, *Prevotella***↓**, *Streptococcus***↑**	19 RRMS/17 HS	^23^
*Bacteroides coprocola***↓**, *Bacteroides coprophilus***↓**, *Bacteroides stercoris***↓**, *Eggerthella lenta***↑**, *Prevotella copri***↓**, *Streptococcus thermophilus***↑**, *Sutterella wadsworthensis***↓**	20 RRMS/40 HS	^24^
*Bacteroides***↓**, *Faecalibacterium***↓**, *Ruminococcus***↑**	5 RRMS/8 HS	^25^
*Bilophila***↓**, *Blautia***↑**, *Butyricicoccus***↓**, *Clostridium XIVb***↓**, *Dorea***↓**, *Faecalibacterium***↓**, *Flavonifractor***↑**,*Gemella***↓**, *Granulicatella***↓**, *Haemophilus***↓**, *Roseburia***↓**	22 RRMS/33 HS	^26^
*Faecalibacterium***↓**	34 RRMS/165 HS	^27^
*Akkermansia***↑**, *Clostridium***↑**, *Prevotella***↓**	40 RRMS, multi-ethnic ancestry/41 HS	^28^
*Prevotella***↓**	13 RRMS/14 HS	^29^
*Coprococcus***↓**, *Lachnospira***↓**, *Roseburia***↓**, *Streptococcus***↑**	26 RRMS/38 HS	^30^
*Akkermansia***↑**, Coprococcus**↓**, Roseburia**↓**	12 SPMS/38 HS	^30^
*Akkermansia***↑**	34 twins pairs MS/HS	^31^
*Akkermansia***↑**, *Blautia wexlerae***↓**, *Clostridium bolteae***↑**, *Dorea formicigenerans***↓**, *Erysipelotrichaceae CCMM***↓**, *Ruthenibacterium lactatiformans***↑**	199 RRMS and 44 progressive MS/40 HS	^32^
*Anaerococcus vaginalis***↓**, *Blautia faecis***↓**, *Clostridium g24 FCEY***↑**, *Dorea longicatena***↓**, *Enterobacteriaceae***↑**, *Ruminococcaceae FJ366134***↑**	44 progressive MS/199 RRMS and 40 HS	^32^
*Akkermansia***↑**, *Clostridium***↑**, *Eubacterium***↓**, *Lachnospiraceae***↓**, *Megamonas***↓**, *Streptococcus***↑**	62 RRMS/55 HS	^33^
*Clostridium***↑**, *Streptococcus***↑**	15 SPMS/55 HS	^33^
*Acinetobacter calcoaceticus***↑**, *Akkermansia muciniphila***↑**	64 MS/64 HS	^34^
*Prevotella***↓**, *Streptococcus***↑**	34 RRMS/34 NMOSD and 34 HS	^35^
*Actinomyces***↑**, *Clostridium III***↑**, *Eggerthella***↑**, *Faecalicoccus***↑**, *Gemmiger***↓**, *Lachnospiraceae***↓**, *Sporobacter***↓**, *Streptococcus***↑**	19 RRMS/21 RA and 20 CD and 19 UC and 23 HS	^36^
*Gemmiger***↑**, *Ruminococcus***↑**	15 PPMS/15 HS	^37^
*Bacteroides***↑**, *Clostridium***↓**, *Coprococcus***↓**, *Firmicutes***↓**, *Methanobrevibacter***↓**, *Paraprevotella***↓**, *Proteobacteria***↓**, *Ruminococcaceae***↓**	26 RRMS/39 HS	^38^

The alterations are indicated by arrows. Abbreviations: Crohn’s disease – CD, healthy subjects – HS, neuromyelitis optica spectrum disorder – NMOSD, rheumatoid arthritis – RA, RRMS – relapsing-remitting multiple sclerosis, SP – secondary progressive multiple sclerosis, PPMS – primary progressive multiple sclerosis, ulcerative colitis – UC.

Further clinical studies were designed to investigate factors contributing to altered composition and function of the gut microbiome in MS.^31,40^ A study which included twins discordant for the disease indicated interaction of environmental and genetic factors in this field.^31^ The International MS Microbiome Study Consortium paired MS subjects with household HS (to minimize the effect of dietary habits) and demonstrated the relationships between the gut microbiome alterations and MS risk and stage of disease.^40,41^ Variability of microbial composition in MS subjects may be further affected by age and gender, dietary components, comorbidities and related therapies (e.g. antibiotics intake).

It is still debatable whether gut dysbiosis can trigger (through the gut-brain axis) the autoimmune response involved in the background of MS, or whether it merely reflects the disturbed balance between pro- and anti-inflammatory components of this autoimmune response. Experimental and clinical studies have been undertaken to elucidate these relationships and reveal their relevant aspects.

### The role of gut dysbiosis in MS pathology

#### Experimental studies

Studies conducted in an animal model of MS – experimental autoimmune encephalitis (EAE) – showed the pattern of alterations in the gut microbiome similar to human studies and related to the course of the disease. Analysis of the intestinal flora at pre-onset, onset, peak and chronic phase of EAE revealed that the abundance of the *Lactobacillaceae* family was decreased, while other populations such as *Clostridiaceae*, *Ruminococcaceae* and *Peptostreptococcaceae* were expanded in the course of the disease.^42^ Gandy et al. found diverse microbial populations characteristic for SJL/J mice having relapsing-remitting EAE (RR-EAE) and C57BL/6 mice with chronic progressive EAE (CP-EAE). In RR-EAE mice there was a significant expansion of *Bacteroidales* (including *Bacteroides*, *Parabacteroides*, *Prevotella*, *Rikenellaceae* and *Odoribacter*), compared to CP-EAE. In turn, CP-EAE mice were identified with significantly elevated *Akkermansia muciniphila*.^43^ Moles et al. evaluated the relationship between microbiome composition and disease symptoms using EAE and the cuprizone demyelination model (the latter characterized by a progressive course).^44^ In both models, an increase of *Firmicutes* and decrease of *Bacteroides* were observed at the onset of clinical symptoms, accompanied by a remarkable decrease in *Actinobacteria*, represented mainly by *Bifidobacterium*. Alterations specific only for the cuprizone model included an apparent rise of the phylum *Verrucomicrobiota*, exclusively represented by *Akkermansia*, as well as the *Sutterella* population, which appeared after cuprizone exposure and during the remyelination process. Furthermore, diversity of microbiota correlated with severity of demyelination and scores for clinical symptoms.

Further experimental studies provided some evidence for a causal link between gut dysbiosis and autoimmune CNS involvement. It was demonstrated that germ-free (GF) mice (lacking the gut microbiota or treated with antibiotics) did not develop EAE or presented with delayed and reduced disease activity.^45^ In turn, the colonization of GF mice with segmented filamentous bacteria resulted in an increased IL-17 level in the gut, which favored Th17 proliferation and ultimately caused development of EAE.^46^ In contrast, monocolonization with *Bacteroides fragilis* prevented EAE in these mice by inducing tolerogenic CD103^+^ dendritic cells (DCs)^47^ and enhanced production of intestinal Tregs secreting IL-10.^48^ This protective effect of *B. fragilis* was attributed to its structural component, polysaccharide A. Another interesting investigation, which involved testing a single probiotic strain – *Lactobacillus helveticus*^49^ – for the EAE effect, also indicated differential modulation of the autoimmune response resulting in EAE attenuation. Examples of other bacteria able to ameliorate EAE include *Lactobacillus* strains (*Lactobacillus crispatus*, *Lactobacillus rhamnosus*, *Lactobacillus paracasei*, *Lactobacillus plantarum* and *Lactobacillus reuteri*)^42,50–52^ as well as *Bifidobacterium* strains (*Bifidobacterium animalis* and *Bifidobacterium bifidum)*. ^53,54^ There are also reports demonstrating that the combination of two probiotic strains – *Bifidobacterium animalis* with *Lactobacillus plantarum*^55^ or *Enterococcus faecium* with *Prevotella histicola*^56^ – ameliorated neuroinflammation in the EAE model. Surprisingly, it has been found that treatment with a probiotic mixture of *Streptococcus thermophilus*, *Lactobacillus reuteri*, *Bifidobacterium bifidum*, *Lactobacillus acidophilus* and *Lactobacillus casei* during induction of EAE delayed disease onset.^54^

CNS infection caused by Theiler’s encephalomyelitis virus (TMEV) – an experimental model of progressive MS – altered the composition of the gut microbiota of SJL/J mice toward moderate dysbiosis at particular phases of the disease. In the acute phase there was a decrease in the *Alloprevotella* population, at the presymptomatic stage a decrease of *Akkermansia* and *Anaerotruncus*, and at the chronic stage a decrease of *Streptococcus*. Conversely, *Clostridium* and *Eubacteria* were increased throughout the disease. Furthermore, TMEV infection was observed to increase intestinal permeability and CD4^+^ T cell count in the lamina propria.^57^ Another study on a murine model of TMEV infection showed its selective impact on gut microbiota, with increased abundance of *Marvinbryantia* and *Coprococcus* genera. Links were identified between these bacterial genera and the CNS transcriptome for the T cell receptor, immunoglobulins, major histocompatibility complex (MHC) and complement genes.^58^

In a cuprizone-induced demyelination model, altered β-diversity of the gut microbiome was found in mice, as well as a positive correlation between relative abundance of some species (including *Eisenbergiella* and *Faecalibaculum*) and extent of CNS demyelination and activation of microglia.^59^ Moreover, subdiaphragmatic vagotomy in cuprizone-treated mice attenuated demyelination and microglial activation in the corpus callosum and also partially restored the abnormal β-diversity of gut microbiota (inter alia with an increase in relative abundance of *Lactobacillus* and *Turicibacter*). These findings suggested the involvement of the vagus nerve (as a component of the gut–brain axis) in pathomechanisms of cuprizone-induced CNS demyelination.^60^

Interesting observations were based on a transfer of intestinal content between human or animal subjects. Transfer of feces obtained from mice with EAE at peak disease activity to naïve mice (before their immunization) suppressed development of the disease in recipients. The feces from EAE animals (as well as untreated pwMS) was shown to be enriched in microRNA-30d, which regulated the expression of lactase essential for growth of *Akkermansia muciniphila*. Increased abundance of *Akkermansia* in the gut was associated with stimulation of Tregs and related cytokines, supposedly contributing to amelioration of CNS inflammation.^61^

Furthermore, administration of *Akkermansia* isolated from pwMS to animals was shown to ameliorate EAE by reduction of RORγt+ and IL-17 produced by γδ T cells.^32^ EAE also developed in GF mice after transplantation of gut microbiota obtained from pwMS, in contrast to those who obtained it from HS.^19,31^ These findings suggest the potential relevance of the microbiota-gut-brain axis in the pathogenesis of MS.

Taken together, the above *in vivo* data indicate that the gut dysbiosis is functionally linked with a shift from regulatory components of the autoimmune response toward pro-inflammatory ones. These outcomes were believed to be affected by microbial metabolites, mainly SCFA fermentation products. It has been shown that SCFA (e.g. propionate) may directly affect T cell subsets, with decreased differentiation of Th17 cells^62^ and increased differentiation of Tregs and their enhanced suppressive capacity.^63^

Moreover, microbial SCFA was shown to influence the BBB permeability^64^ and the CNS resident cells like microglia.^65^ GF mice, compared to those with normal gut microbiota, possessed reduced expression of tight junction proteins, which lead to increased BBB integrity. The BBB permeability could be modulated by SCFA-producing bacteria or directly by their metabolites, such as butyrate.^64^ GF mice also presented abnormal maturation and differentiation of microglial cells; their function could be restored using SCFA metabolites or following colonization with microbiota capable of SCFA production.^65^

#### Clinical evidence

The aforementioned findings from experimental studies supported further thorough clinical investigation of the gut microbiome in pwMS, considering the more complex nature of the disease compared to an animal model, with additional confounding factors.

In RRMS patients, an increase in *Akkermansia muciniphila*, a mucin degrading bacteria, and *Acinetobacter calcoaceticus* was observed, and they were found to provoke pro-inflammatory activity *in vitro*.^19^ Particularly, MS-derived *Akkermansia muciniphila* enhanced differentiation of human T cells toward Th1 cells,^19^ while *Prevotella* (otherwise decreased in MS subjects)^66^ enhanced cell differentiation toward Th17 expansion. In addition, Jangi et al. reported that increased *Akkermansia* and *Methanobrevibacter* positively correlated with the expression of genes for pro-inflammatory T cells and monocytes, both implicated in the pathogenesis of MS.^67^ Furthermore, elevated antibodies against *Acinetobacter* in serum of MS patients suggest a possible role of this bacteria in autoimmune cross-reactivity.^68^ Another example linking microbial alteration with a dysregulated immune response in MS is frequently identified depletion of *Bacteroides*,^19–25^ associated with a lower level of lipid 654, a TLR2 ligand, relevant for innate immunity.^69^ Cekanavicute et al. found that a particular *Bacteroides* species, *Parabacteroides distasonis*, can augment the function of Tregs *in vitro*. Thus, the lower abundance of this species may result in suppressed immunoregulatory activity.^34^ Furthermore, *Bacteroides fragilis* was found to modulate maturation of the immune system through bacterial polysaccharide, by correcting deficiencies of T cells and Th1/2 imbalance, and eliciting production of appropriate cytokines.^70^

Findings from clinical studies also indicated some metabolic alterations potentially related to the gut microbiome. Reduced activity of microbial SCFA producers was found in RRMS patients, as well as a decreased level of their metabolite butyric acid, in contrast to an upregulated level of caproic acid (medium chain FA with pro-inflammatory properties). Absence of the butyrate-producing *Fusobacteria* or *Methanobrevibacter*^38^ was associated with a shorter time to MS relapse.^71^

Functional differences in gut microbiota were demonstrated for various phenotypes of MS.^33^ As indicated above, in RRMS patients a decrease in regulatory bacteria, such as *Parabacteroides* and *Prevotella* (*Bacteroidetes*), *Adlercreutzia* and *Collinsella* (*Actinobacteria*) as well as *Erysipelotrichaceae* (*Firmicutes*), has been noted.^66^ *Adlercreutzia, Parabacteroides* and *Prevotella* can process phytoestrogens into monomeric compounds, decreasing chemo-attracting proteins-1 and IL-6, thus diminishing oxidative stress and inflammatory cytokine activity.^72^ In turn, *Erysipelotrichaceae* play an important role in bile acid metabolism and ensure homeostasis at the mucosal surface.^73^ In contrast to RRMS subjects, those with SPMS were characterized by elevated levels of oxidative stress markers such as: i) an increase of microbial genes involved in mismatch DNA mutation repair and ii) an increased ratio of cysteine persulfide to cysteine, indicating excessive DNA oxidation.^33^ In addition, the presence of *Clostridium* strains in both RRMS and SPMS was associated with higher scores of disability and fatigue.^33^

## Mechanistic concepts of the gut microbiome contribution to MS background

The experimental and clinical evidence revealed the links between gut dysbiosis in MS and the dysregulated immune response, as well as altered metabolic pathways, corresponding to the clinical course of the disease. The key points of this investigation included: i) imbalance between pro-inflammatory and anti-inflammatory/regulatory components of the immune response involved in the MS background and ii) ability of commensal bacteria to promote either the pro- or anti-inflammatory pathway via different mechanisms.^74^

### Impact upon the autoimmune response and disease induction

Among potential mechanisms of the gut microbiome triggering CNS inflammation, the relevant evidence indicates molecular mimicry – cross-reactivity between self-antigens of CNS and microbial peptides. Some similarities were found between myelin basic protein (MBP) and proteins of *Bacteroides* and *Bifidobacterium* species. Homologous reactivity of CD4^+^ T cell clones has also been demonstrated for epitopes of MBP and GDP-L-fucose synthase of *Akkermansia* and *Prevotella*. Effects of bacteria from the *Erysipelotrichaceae* family and *L. reuteri* on EAE have been associated with myelin oligodendrocyte protein (MOG) molecular mimicry.^75,76^

Furthermore, bacterial products (e.g. peptidoglycan or N-acetylmuramyl dipeptide) have been found to stimulate receptors on antigen-presenting cells, triggering production of pro-inflammatory mediators and promoting bystander lymphocyte activation.

Other pathogenic mechanisms of the gut microbiome affecting CNS autoreactive inflammation might be associated with its direct impact on immunocompetent cells. Particular microbes (e.g. segmented filamentous bacteria) are able to induce differentiation of Th17 cells in the ileum. Pro-inflammatory properties of these cells may be activated by presence of some dietary compounds (salt or long chain fatty acids). After migration to lymph nodes, Th17 cells can lower the threshold for activation of autoreactive T cells, promoting an autoimmune response.

Interestingly, protective anti-inflammatory effects have been observed for other bacteria (*Clostridia*, *Bacteroides*, *Prevotella*). These effects were suggested to be associated with their particular components, such as polysaccharide A and lipid 654, a TLR2 ligand. The relevant role of these molecules includes an influence on Treg differentiation in the colon, promoting the expansion of FoxP3^+^ Tregs, correcting Th1/2 imbalance, stimulating production of anti-inflammatory cytokines, and induction of tolerogenic DCs and suppressive macrophages.^10,47,48,70,75^

### Impact upon metabolic pathways and their links with immune and CNS system

The gut microbiome metabolites take part in a range of metabolic pathways relevant for inflammatory and neurodegenerative processes in the CNS.

#### SCFAs

**Figure 2.**
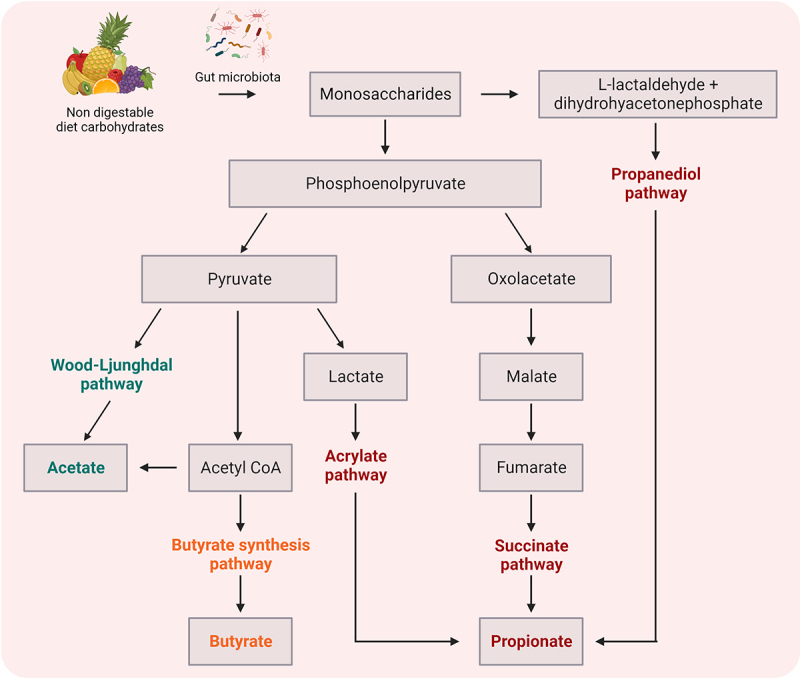
Methabolic pathways of bacterial fermentation resulting in the production of short chain fatty acid (SCFA) components.

The SCFAs, by-products of the fermentation of complex and indigestible carbohydrate in the colon, i.e., acetate (C2), propionate (C3) and butyrate (C4) (Figure 2), are known to be potent immune modulators. Furthermore, SCFAs are able to cross the BBB, which is associated with expression of: i) proton or sodium-dependent monocarboxylate transporters (MCT and SMCT, respectively) and ii) various receptors including extracellular G protein coupled receptors (GPR), namely free fatty acid receptors (FFAR), broadly expressed in various tissues and immune cell types.^77^ These findings imply the direct influence of SCFAs on the function of CNS resident cells affecting homeostasis (Table 2). The main processes triggered by SCFAs included activation of FFAR2 and FFAR3, also known as GPR43 and GPR41.^77^ The SCFAs activate ERK,^78^ JNK,^62,78^ p38-MAPK.^62^ NRF2^81^ and NF-κB^78^ signaling pathways, which modulate the activity of both innate and adaptive immune cells. It has also been observed that SCFA metabolites – acetate, propionate and butyrate – promote IL-10 and FoxP3 peripheral production^88^ and inhibit TNF-α and IL-1β in the CNS.^90^ In addition, after entering the cells, SCFAs can inhibit histone deacetylase (HDACs)^80,82,84,86^ and directly influence gene expression, causing among other things upregulation of regulatory mediators (IL-10, FoxP3)^63,80,87,88^ or downregulation of pro-inflammatory mediators (iNOS, IL-6 and IL-12).^84^

**Table 2.** Mechanisms of action of bacterial fatty acid metabolites on events related to MS background.

Fatty acid metabolite(s)	Effect on	Proposed mechanism	Research model	Reference
Formate, acetate propionate, butyrate, pentanoate	Various tissues and mammalian cells	Activation of FFAR2 and FFAR3	*In vitro*	^77^
Acetate	Microglia	Upregulation of FFAR3 and suppression of the ERK/JNK/ NF-κB pathway	*In vitro* and *in vivo*	^78^
Acetate	Lipids	Increased acetyl-CoA metabolism alters fatty acid metabolism and gangliosides GD3 and GD1a content, prevents the loss of myelin and cholesterol (but not cholesteryl esters) and restores the level of cytosolic phospholipase A2 (cPLA2)	*In vivo*	^79^
Propionic acid	T cells	Suppressive capacity through IL-10 producing Tregs and elevated mitochondrial respiration	*In vitro* and *ex vivo*	^63^
Propionate	T cells	Induced HDAC inhibition increases IL-10 and FoxP3 expression in an FFAR2-dependent manner	*In vivo*	^80^
Propionate	BBB	Inhibition of pathways associated with nonspecific microbial infections via a CD14-dependent mechanism, suppression of expression of LRP-1 and protection of the BBB from oxidative stress via Nrf2 (NFE2L2) signaling	*Ex vivo* and *in vitro*	^81^
Butyrate	T cells	Induced expansion of Tregs by enhanced histone H3 acetylation in the promoter and conserved non-coding sequence regions of the FoxP3 locus	*In vivo*	^82^
Butyrate	T cells	Facilitation of TGF-β1-dependent generation of FoxP3+ Tregs, induction of expression of T-bet and IFN-γ as well as induction of H3 acetylation at tbx21 and Ifnγ locus during their differentiation, and promotion of Th1 polarization in an FFAR2, FFAR3 and SMCT1-independent manner	*In vivo*	^83^
Butyrate	Macrophages	Downregulation of pro-inflammatory mediators that included iNOS, IL-6 and IL-12 – effects mediated by inhibition of histone deacetylases	*In vitro* and *in vivo*	^84^
Butyrate	Oligodendrocytes	Induced suppression of demyelination and enhancement of remyelination; these effects were independent of microglia and were likely mediated by their activity as a HDAC inhibitor	*In vivo*	^85^
Butyrate, propionate and valerate	T cells	A boost in Tregs upon provision of butyrate due to potentiation of extrathymic differentiation of Tregs dependent on intronic enhancer of conserved non-coding sequence 1 and potentiated by propionate de novo Treg generation in the periphery, another SCFA capable of histone deacetylase inhibition	*In vivo*	^86^
Valerate/pentanoate	T cells and B cells	Elevated glucose oxidation mediated by pentanoate-triggered Akt/mTOR signaling pathway (increased IL-10 production by Bregs and effector T cells) along with its HDAC-inhibitory activity (reduced IL-17A expression)	*In vivo*	^87^
SCFAs	BBB permeability	Increased expression of the tight junction proteins occludin and claudin-5	*In vivo*	^64^
SCFAs	T cells	Expansion of gut Tregs by suppression of the JNK1 and p38-MAPK pathway	*In vivo*	^62^
SCFAs	T cells	Bidirectional regulatory potentials driven by: 1) induction of IL-10-producing T cells and 2) promotion the expression of IL-10 by conditioning CNS-resident APCs	*In vitro* and *in vivo*	^88^
SCFAs	Microglia	Downregulation of FFAR2 is responsible for microglia defects	*In vivo*	^89^
SCFAs	Microglia-like cells	Decreased secretion of IL-1β, MCP-1, TNF-α and cytotoxins (an effect not mediated by FFAR2/3) as well as reduced phagocytic activity and production of ROS	*In vitro*	^90^
SCFAs	BBB permeability	Reduced expression of the tight junction proteins occludin and claudin-5	*In vivo*	^64^
LCFAs	T cells	Enhanced differentiation and proliferation of Th1 and/or Th17 cells and their impaired intestinal sequestration via p38-MAPK pathway	*In vivo*	^62^

Abbreviations: Akt – serine/threonine protein kinase, APC – antigen presenting cell, BBB – blood–brain barrier, CNS – central nervous system, cPLA_2_ – cytosolic phospholipase A_2_, ERK – extracellular signal-regulated kinase, FFAR – free fatty acid receptor, HDAC – histone deacetylase, iNOS – induced nitric oxide synthase, JNK – Jun N-terminal kinase, LCFA – long chain fatty acid, MAPK – mitogen-activated protein kinase, MCP-1 – monocyte chemoattractant protein 1, mTOR – mammalian target of rapamycin, NFE2L2 –nuclear factor erythroid‐derived 2‐like 2, NFκB – nuclear factor kappa light chain enhancer of activated B cells, Nrf2 – nuclear factor erythroid 2-related factor 2, ROS – reactive oxygen species, SCFA – short chain fatty acid, SMCT1 – sodium-coupled monocarboxylate transporter 1.

SCFAs were also reported to maintain and increase BBB integrity by increased expression of the tight junction proteins occludin and claudin-5.^64^ PwMS who had a lower butyric acid (BA)/caproic acid (CA) ratio showed higher intestinal permeability and more Th1 cells. The increase in the BA/CA ratio correlated positively with Treg subsets and negatively with IFN-γ-producing lymphocytes.^30^

Moreover, acetate-mediated fatty acid synthesis in oligodendrocytes and HDAC-dependent butyrate action are believed to contribute to protection of CNS oligodendroglia, with suppressed demyelination^79^ and enhanced remyelination.^85^

#### Polyunsaturated fatty acids

Polyunsaturated fatty acids (PUFAs) are characterized by the presence of multiple double bonds within the fatty acid chain, which determines their properties. Particular interest has been attributed to omega-3 PUFA, e.g. α-linolenic acid (ALA), eicosapentaenoic acid (EPA) and docosahexaenoic acids (DHA). Omega-3 PUFA can alter the diversity and abundance of gut microorganisms, particularly influencing beneficial bacteria such as *Bifidobacterium* and *Akkermansia*. In addition, they improve the intestinal mucosal barrier function by increasing its thickness and reducing its damage caused by inflammatory and oxidative processes (e.g. LPS, hydrogen peroxide, increased mitochondrial activity).^91^ More importantly, omega-3 PUFA regulate gut homeostasis and its immunity. They are known to modulate intestinal immunity via the nuclear transcription factor κB (NF-κB) and MAPK signaling pathways^92^ as well as the metabolic pathway of arachidonic acid.^93^ These acids also possess the ability to control the level of pro-inflammatory (e.g. endotoxins and IL-17) as well as anti-inflammatory (e.g. SCFAs and their salts) mediators.^94^

#### Tryptophan metabolites

**Figure 3.**
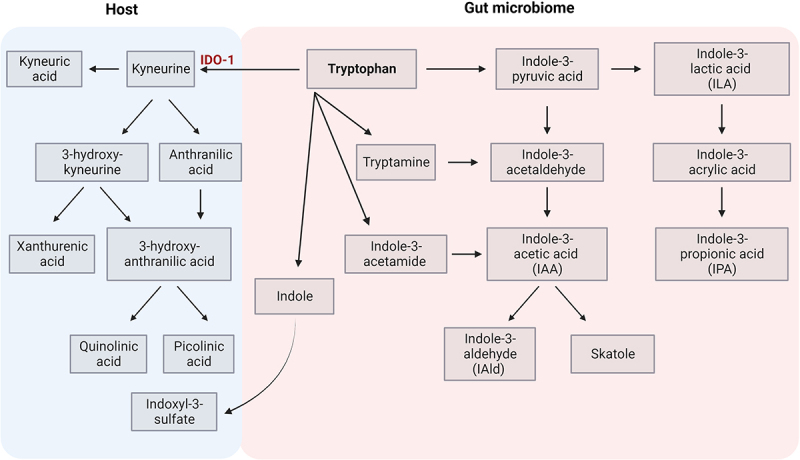
Tryptophan metabolic pathways.

Tryptophan is an essential amino acid metabolized by the gut microbiota into indole-containing compounds. Particularly, *Lactobacillus* strains were shown to directly utilize tryptophan as a substrate and metabolize it into indole-3-lactic acid (ILA), indole-3-acetic acid (IAA) and indole-3-aldehyde (IAld) in the gut.^95^ Moreover, tryptophan itself can be transported from the gut into the circulation and metabolized by the kynurenine and serotonin pathway (Figure 3). Both tryptophan and its metabolites can act as potent immunomodulators by binding to the aryl hydrocarbon receptor (AhR) (Table 3). Different levels of circulating AhR ligands were found in RRMS patients, depending on the disease activity.^96^

Only tryptophan catabolism via the indole and kynureine pathways have been shown; serotonic pathway has been excluded. Indole pathway takes place in gut lumen via gut microbiome although tryptophan could be also converted to kyneurine in the host via indolamine 2,3-dioxygenase (IDO-1).

**Table 3.** Mechanisms of action of tryptophan metabolites on events related to MS background.

Tryptophan metabolite(s)	Effect on	Proposed mechanism	Research model	Reference
I3C, indole, I3S, indirubin, kynurenine	AhR ligands	Decreased levels of circulating AhR ligands in RRMS compared to active MS or benign MS	*Ex vivo*	^96^
IPA, I3C	T cells	Dietary tryptophan mediated disease protection via microbiota-dependent impairment of autoreactive T cells	*In vivo*	^97^
IAld	T cells	Suppression of Th17 polarization and IL17 production	*In vivo*	^98^
IAld	Mucosal surface	Induction of mucosal IL-22 via AhR	*In vivo*	^99^
IPA	Mucosal surface	Regulation of mucosal integrity via PXR dependent manner and TLR4 signaling	*In vivo*	^100^
I3S, IPA and IAld	Astrocytes	I3S, IPA and IAId mediated limitation production of Il-6, TNF-α, CCL2 and iNOS in astrocytes via AhR signaling, resulting in MS amelioration	*In vivo*	^101^
I3S	Microglia and astrocytes	Dietary tryptophan metabolites control microglial activation and TGF-α and VEGF-B production and modulation astrocyte activities via AhR	*In vivo and* *in vitro*	^102^
Kynurenine metabolites	BBB	Basolateral secretion of kynurenine (by BBB endothelial cells) further metabolized to quinolinic acid, leading to neurotoxic effect	*In vitro*	^103^
Kynurenine metabolites	T cells	Suppressive effect of T cell mediated by IDO-1 resulting in more chronic form of kynurenine pathway activation, leading to MS progression by production of excitotoxic quinolinic acid and increased quinolinic acid/kynurenic acid ratio by infiltrating macrophages	*Ex vivo*	^104^
Quinolinic acid	Microglia	Detectable amount of quinolinic acid production by microglia, lack of production of quinolinic acid by neurons	*In vitro*	^105^
Laquinimod	Astrocytes	Amelioration of the disease via AhR activation in astrocytes by laquinimod	*In vivo*	^106^

Abbreviations: AhR – aryl hydrocarbon receptor, BBB – blood-brain barrier, CCL2–chemokine (C-C motif) ligand 2, I3C – indole-3-carbinol, I3S – indole-3-sulfate, IAld – indole-3-aldehyde, IDO-1–indolamine 2,3-dioxygenase, iNOS – induced nitric oxide synthase, IPA – indole-3-propionic acid, PXR – pregnane X receptor, RRMS – relapsing-remitting multiple sclerosis, TLR – toll-like receptor, VEGF-B – vascular endothelial growth factor B.

Tryptophan metabolites may modulate T cell subsets’ differentiation by either promoting Th1/Th17 cells or generating Tregs.^97,107^ IAld was found to inhibit Th17 polarization, to reduce production of IL17^98^, and to induce mucosal IL-22 via AhR.^99^ A favorable effect of *Lactobacilli* on EAE outcome was attributed to properties of this metabolite.^51,108^ Indole and indole-3-propionic acid (IPA) have also been reported to promote tight junction formation, acting directly on epithelial cells in a pregnane X receptor (PXR) dependent manner.^100^

Metabolic products of the kynurenine pathway can affect brain epithelial cells depending on the condition. Basically, endothelial cells convert tryptophan to kynurenic acid. However, in the presence of pro-inflammatory cytokines, TNF-α and IFN-γ, endothelial cells express indolamine 2,3-dioxygenase (IDO-1), which converts tryptophan to kynurenine, further metabolized by perivascular macrophages and microglia to quinolinic acid.^103^ Production of quinolinic acid by activated microglia and infiltrating macrophages has been reported to have neuroinflammatory and neurodegenerative effects.^105^ Increased quinolinic acid/kynurenic acid ratio in patients with progressive types of MS may suggest that quinolinic acid is relevant for the neurodegenerative component of the disease background.^104^ On the other hand, indole-containing metabolites, namely indole-3-sulfate (I3S), IPA and IAld, limit production of Il-6, TNF-α, CCL2, and iNOS from astrocytes, resulting in disease amelioration.^101^ In addition, it has been shown that dietary tryptophan metabolites control microglial activation and TGF-α and VEGF-B production, and in consequence modulate astrocyte activities via AhR.^102^ Amelioration of disease in animal models of MS by synthetic indole derivatives was suggested to be mediated by AhR signaling in astrocytes.^106^

Apart from these main metabolic pathways involving tryptophan (AhR and PXR ligands), there is also some evidence for relevance of other ones (e.g. tryptophan-derived serotonin)^109^ in the links between the gut microbiome and MS, which require further investigations.

#### Polyamines

**Figure 4.**
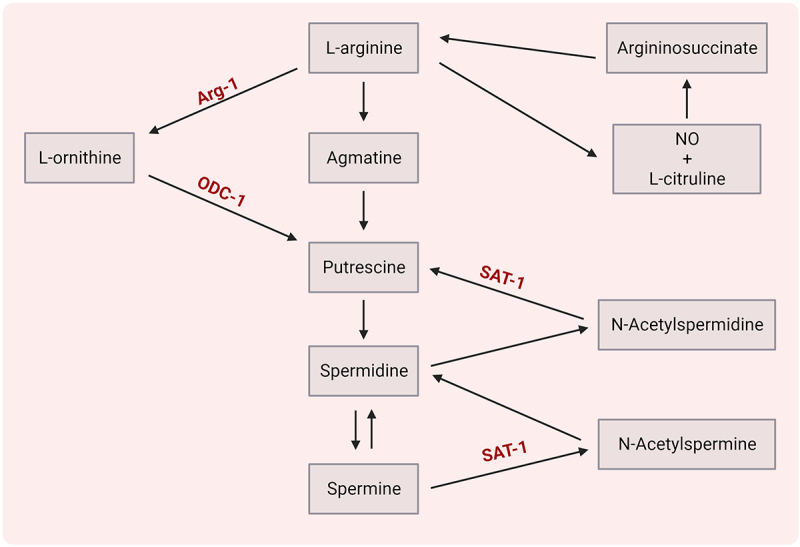
Polyamine metabolism.

Abbreviations: Arg-1 – arginase 1, ODC-1 – ornithine decarboxylase 1, SAT-1 – spermidine/spermine N1 acetyltransferase 1.

Polyamines (putrescine, spermidine and spermine), derived from the L-arginine metabolic pathway (Figure 4), can be produced either by the host or by gut microbiota (i.e. *Bacteroides thetaiotaomicron*, *Fusobacterium varium*, *Enterococcus*, *Bifidobacterium*).

These compounds can diminish neuroinflammation by modulation of T cells or modulation of microglia/macrophages (Table 4). Spermidine was found to shift the polarization of Th17 cells toward Tregs in the murine gut in a TGF-β-dependent manner.^111^ Strikingly, suppression of ornithine decarboxylase 1 (ODC-1) or spermidine/spermine N1 acetyltransferase 1 (SAT-1) – enzymes involved in polyamine metabolism – can specifically limit Th17 function in a putrescine-dependent mode.^110^

**Table 4.** Mechanisms of action of polyamine metabolites on events related to MS background.

Polyamine metabolite(s)	Effect on	Proposed mechanism	Research model	Reference
Putrescine	T cells	Suppression of ODC1 or SAT1 restricts Th17 function in a putrescine-dependent manner	*In vitro*	^110^
Spermidine	T cells	Shift of polarization of Th17 cells toward FoxP3+ Treg differentiation in a TGF-β dependent manner	*In vitro* and *in vivo*	^111^
Spermidine	Macrophages and T cells	Synergistic mode of action on activation and proliferation of T cells by: i) suppression of expression of pro-inflammatory cytokines IL-1β, IL-12 and co-stimulatory molecules CD80 and CD86 in macrophages through downregulation of activity of NF-κB pathway and ii) upregulation of expression of Arg-1 in macrophages, both causing a shift to M2 phenotype	*In vivo*	^112^
Spermidine	Astrocytes	Suppression of astrocyte-derived chemokines (MIP-1α, MCP-1, RANTES)	*In vivo*	^113^
Spermidine, spermine	T cells	Suppression of LFA-1 expression on human T lymphocytes	*Ex vivo* and *in vitro*	^114^

Abbreviations: Arg-1 – arginase 1, LFA-1 – lymphocyte function associated antigen 1, MCP-1 – monocyte chemoattractant protein-1, MIP-1α – macrophage inflammatory protein-1α, NFκB – nuclear factor kappa light chain enhancer of activated B cells, ODC-1 – ornithine decarboxylase 1, RANTES – regulated on activation, normal T cell expressed and secreted, SAT-1 – spermidine/spermine N1 acetyltransferase 1.

Following treatment with spermidine, amelioration of EAE was observed, associated with decreased secretion of pro-inflammatory cytokines IL-1β and IL-12, lowered levels of co-stimulatory molecules CD80 and CD86 by inhibition of the NF-κB pathway along with upregulation of expression of arginase 1 (Arg-1) in macrophages^112^, both causing a shift to M2 phenotype. Spermine and spermidine were also reported to reduce immune cell infiltration into the CNS, as a result of decreased release of astrocyte-derived chemokines, such as MIP-1a, MCP-1, and RANTES^113^ or direct inhibition of LFA-1 on T cells.^114^

#### Polyphenols

Polyphenols can be classified into flavonoids (e.g. apigenin, epigallocatechin-3-gallate, hesperidin A) and nonflavonoids (e.g. resveratrol). They exert a protective effect on neurons by acting against oxidative stress and suppressing pro-inflammatory NF-κB pathway. The gut microbiota contributes to the biotransformation of polyphenols into their bioavailable forms and active metabolites, relevant for the synthesis of neurotransmitters.

Ellagic acid, another polyphenol compound, is metabolized in the gut by *Gordonibacter urolithinfaciens* and *Gordonibacter pamelaeae* and generates urolithins. Studies have shown that urolithins exert effects on T cells, DCs, microglia, oligodendrocytes and neurons (Table 5). Interestingly, ellagic acid, a urolithin A precursor, has been shown to protect against myelin-associated sphingolipid loss by affecting ceramide synthesis.^120^ In addition, Zhang et al. reported that T cell activation and proliferation can be suppressed by urolithin A through interfering with calcium machinery in a miR-10a-5p dependent manner.^115^ In line with this, reduced infiltration of Th1/Th17 cells and monocytes to the CNS by targeting AhR was observed.^116^ The impact of urolithin A administration also included reduced levels of co-stimulatory molecules CD80, CD86 and MHC-II on DCs, as well as a lower proportion of M1 pro-inflammatory type of microglia.^116^

**Table 5.** Mechanisms of action of urolithin metabolites on events related to MS background.

Urolithins metabolite(s)	Effect on	Proposed mechanism	Research model	Reference
Urolithin A	T cells	Suppression of T cell activation and proliferation by modulating calcium machinery (downregulation of Orai1/STIM1/2 expression) in a miR-10a-5p-dependent manner	*In vivo*	^115^
Urolithin A	T cells, DCs, and microglia	Reduced levels of CD80, CD86 and MHC-II on DCs, lower numbers of M1 type microglia, activated DCs as well as reduced infiltration of Th1/Th17 cells and monocytes by targeting AhR and modulating the signaling pathways	*In vitro* and *in vivo*	^116^
Urolithin A	Gut barrier integrity	Upregulation of epithelial tight junction proteins through AhR and Nrf2 dependent pathways	*In vitro* and *in vivo*	^117^
Urolithin A	Neurons	Neuroprotection and anti-inflammatory effect by activation/phosphorylation of the AMPK, p65NFκB, p38MAPK and BACE1 signaling pathways	*In vivo*	^118^
Urolithin A and urolithin B	Microglia and neurons	Decreased secretion of pro-inflammatory markers (NO, IL-6, prostaglandin E2, TNF-α), mitigated apoptosis and caspase 3/7 and 9 release	*In vitro*	^119^
Urolithin A and urolithin B	Oligodendrocytes	Prevention of myelin associated sphingolipid loss by stimulation of synthesis of ceramide	*In vitro* and *in vivo*	^120^
Urolithin B	Neurons	Neuroprotection by suppression of c-JNK activation and cytochrome c release as well as activation of ERK and PI3K pathway along with Akt and p44/42 MAPK phosphorylation and activation	*In vivo*	^121^

Abbreviations: AhR – aryl hydrocarbon receptor, Akt – serine/threonine protein kinase, AMPK – adenosine monophosphate-activated protein kinase, BACE1 – β-site amyloid precursor protein cleaving enzyme 1, DC – dendritic cell, ERK – extracellular signal-regulated kinase, JNK – Jun N-terminal kinase, MAPK – mitogen-activated protein kinase, MHC-II – major histocompatibility complex class II, NFκB – nuclear factor kappa light chain enhancer of activated B cells, Nrf2 – nuclear factor erythroid 2-related factor 2, Orai1 – calcium release-activated calcium modulator 1, PI3K – phosphoinositide 3-kinase, STIM – stromal cell interaction molecule.

Moreover, urolithin A was shown to decrease the epithelial intestinal permeability and attenuate inflammation in an Ahr-Nrf2-dependent fashion through modulation of tight junction proteins.^117^ The neuroprotective effect of urolithins was additionally associated with AMPK, MAPK, JNK, ERK signaling pathways.^118,119,121^

Gut bacteria and their products can spread more easily and become exposed to the immune system due to disruption of the intestinal barrier (“leaky gut” phenomenon). This may be associated with loss of tolerance to bacterial antigens and triggering a local immune response as well as generalized autoimmune activity. Pro-inflammatory cytokines (e.g. IFN-γ and IL-17) may affect function of the intestinal tight junctions and contribute to disruption of the gut barrier. As stated above, intestinal integrity may also be compromised by metabolic alterations (e.g. decreased level of PUFA or increased level of urolithin). Overall, “leaky gut” may result from intestinal dysbiosis but also enhance its consequences, which highlights the complexity and reciprocal character of the links between the gut microbiome and inflammatory processes.^8,75,76^

## Impact of disease-modifying treatment on the gut microbiome in MS

According to current therapeutic standards, immediately after establishing a definite diagnosis of MS, DMT should be initiated, aimed at gaining control over MS activity and sustaining the stable condition of the patients. Due to significant progress in the knowledge about MS background, currently several DMTs are available, differing in efficacy and immunomodulatory/immunosuppressive properties and thus enabling individualized therapeutic strategies. However, DMTs are mainly appropriate for RRMS and targeted at inflammatory processes, while treatment of the progressive phase of MS, including neuroprotective and remyelinating effects, still remains a challenge. Therefore, there is an ongoing investigation in this field, in search for supportive or novel therapeutic options.

In the already mentioned large study comprising MS patients paired with household HS^41^, the composition of the gut microbiome in pwMS differed between the untreated ones and those receiving DMT. Some of the alterations in bacterial taxa found in the untreated MS subgroup in comparison with HS (including *Parabacteroides* and *Akkermansia* species) were not replicated in the subgroup treated with DMT. Furthermore, the use of DMTs was associated with changes in microbial species which did not differentiate untreated MS subjects from controls (e.g. reduction in *Bacteroides*, *Clostridium*, *Roseburia*, *Prevotella* and *Blautia* species and increase in *Phascolarctobacterium* and *Eubacterium*).

The effects of particular DMTs on the gut microbiome have been studied before but usually in small and/or heterogeneous groups (Table 6). Diversity in DMT mode of action and way of administration (injectable or oral) must be considered.

**Table 6.** Modulation of the gut microbiota in MS patients after specified disease-modifying treatment.

Observations noted	Identified in/Compared to	Treatment	Reference
Differences in: *Bacteroidaceae*, *Clostridium*, *other Clostridiales, Faecalibacterium*, *Lactobacillaceae*, *Ruminococcus*	5 RRMS DMT treated / 2 RRMS untreated	5 RRMS GA treated, 2 RRMS untreated	^25^
*Bifidobacterium***↓**, *Faecalibacterium***↑**	36 RRMS DMT treated / 165 HS	27 RRMS DMF treated, 3 RRMS GA treated, 3 RRMS peginterferon-β1a treated, 2 RRMS IFN-β1b treated, 1 RRMS IFN-β1a treated	^27^
*Alistipes***↑**, *Anaerotruncus***↑**, *Butyricicoccus***↓**, *Clostridium cluster IV***↑**, *Gemminger***↓**,*Intestinibacter***↓**, *Lactobacillus***↑**, *Methanobrevibacter***↑**, *Olsenella***↑**, *Parabacteroides***↑**, *Roseburia***↓**, *Ruminococcus***↑**, *Sporobacter***↑**	98 MS (including 26 PPMS, 20 benign MS, 24 active untreated RRMS, 24 RRMS DMT treated, 4 RRMS in relapse / 120 HS	24 RRMS IFN-β treated	^39^
*Butyricicoccus***↓**	26 PPMS / 72 MS (including 20 benign MS, 24 active untreated RRMS, 24 RRMS DMT treated, 4 RRMS in relapse)	24 RRMS IFN-β treated	^39^
*Akkermansia muciniphila***↑**, *Bacteroides finegoldii***↓**, *Blautia***↓**, *Eisenbergiella tayi***↑**, *Faecalibacterium prausnitzii***↓**, *Hungatella hathewayi***↑**,*Roseburia faecis***↓**, *Ruthenibacterium lactatiformans***↑**	576 MS (367 DMT treated, 209 MS untreated) / 576 household HS	71 MS FTY720 treated, 86 MS DMF treated, 68 MS GA treated, 87 MS IFN treated 28 MS anti-CD20 antibody treated, 27 MS NZ treated, 209 MS untreated	^41^
*Adlercreutzia***↓**, *Blautia***↑**, *Collinsella***↓**, *Dorea***↑**, *Haemophilus***↑**, *Lactobacillus***↓**,*Mycoplana***↑**, *Parabacteroides***↓**, *Prevotella***↓**, *Pseudomonas***↑**	31 RRMS (20 RRMS DMT treated, 11 RRMS untreated) / 36 HS	14 IFN-β treated, 1 GA treated, 5 NZ treated 11 untreated	^66^
*Akkermansia***↑**, *Butyricimonas***↓**, *Methanobrevibacter***↑**	60 RRMS patients (32 RRMS DMT treated, 28 RRMS untreated) / 43 HS	14 RRMS GA treated, 18 RRMS IFN-β treated, 28 RRMS untreated	^67^
*Prevotella***↑**, *Sarcina***↓**, *Sutterella***↑**	32 RRMS DMT treated / 28 RRMS untreated	14 RRMS GA treated, 18 RRMS IFN-β treated, 28 RRMS untreated	^67^
Absence of *Fusobacteria phylum*	17 RRMS (9 RRMS DMT treated, 8 RRMS untreated)	5 RRMS GA treated, 3 RRMS IFN-β treated, 1 RRMS NZ treated, 8 RRMS untreated	^71^
Differences in: *Actinobacteria*, *Firmicutes*, *Lentisphaerae*, *Proteobacteria, Prevotella copri* in untreated MS vs. HS**↓,** *Prevotella copri* in treated MS vs. untreated**↑**	30 RRMS / 14 HS	15 RRMS IFN-β treated, 15 RRMS untreated	^122^
*Clostridiales***↓**, *Firmicutes***↓**, *Fusobacteria***↓**, *Lachnospiraceae***↑**, *Veillonellaceae***↑**	93 RRMS DMT treated / 75 RRMS untreated	33 RRMS DMF treated, 60 RRMS GA treated	^123^
*Atypical E. coli***↑**, *Enterobacter* sp.**↑**, *Normal E. coli***↓**	34 RRMS DMT treated	17 RRMS GA treated, 17 RRMS FTY720 treated	^124^
*Bacteroidaceae***↓**, *Bacteroides fragilis***↓**, *Bifidobacterium***↑**, *Bilophila***↑**, *Butyricimonas***↓**, *Christensenellaceae***↑**, *Desulfovibrio***↑**, *Faecalibacterium***↓**, *Lachnospiraceae***↓**, *Methanobrevibacter***↑**, *Ruminococcaceae***↓**	18 RRMS (9 RRMS DMT treated, 9 RRMS untreated) / 17 HS	3 IFN-β treated, 5 GA treated, 1 NZ treated 9 untreated	^125^
Several associations between immune markers and certain gut microbiota including *Bacteroidetes* and *Actinobacteria* were observed	15 RRMS (7 RRMS DMT treated, 8 RRMS untreated) / 9 HS	2 RRMS IFN-β treated, 5 RRMS GA treated, 8 RRMS untreated	^126^

The alterations are indicated by arrows. Abbreviations: DMF – dimethyl fumarate, FTY720 – fingolimod, GA – glatiramer acetate, healthy subjects – HS, NZ – natalizumab, PPMS – primary progressive multiple sclerosis, RRMS – relapsing-remitting multiple sclerosis.

Treatment with IFN-β was associated with lower microbial richness, especially decreased presence of *Ruminococcus* sp., *Clostridium* sp., *F. prausnitzii*, *Roseburia*, but with a concomitant increase in *Parabacteroides distasonis* and *Bacteroides uniformis*.^39,41^ Increased abundance of *Prevotella* (comparable to HS) has also been occasionally noted. Interestingly, administration of *Prevotella histicola* to transgenic mice suppressed EAE as effectively as IFN-β, while the combination of the probiotic and the drug did not further increase the effectiveness of treatment.^122,127^ A similar effect on abundance of *Prevotella* was observed in MS patients treated with glatiramer acetate (GA).^127^ In the experimental model, administration of GA to the EAE mice was associated with an increase in gut *Prevotella*, and combined treatment with the probiotic and GA attenuated the disease more than GA alone.^25^ In clinical studies, treatment with GA was linked with decreases in *Sutterella* and two *Clostridial* families – *Lachnospiraceae* and *Veillonellaceae*^123^ – as well as increases in atypical forms of *E. coli*, *Enterobacter*, *Proteus*, and *Parvimonas micra*.^124^

Similarly to the GA effect, decreased abundance of *Lachnospiraceae* and *Veillonellaceae* was observed during treatment with dimethyl fumarate (DMF), additionally accompanied by a decrease in *Firmicutes* and *Fusobacteria* and an increase in *Bacteroidetes*.^123^ DMF was also found to specifically reduce *Bacteroides stercoris*, *Clostridium*, and *Eubacterium* species,^41^ and tended to normalize the low abundance of *Faecalibacterium* level^124^ and short-term depletion of *Bifidobacterium*,^27^ though these effects were not consistent throughout the studies. Furthermore, DMF and agents with similar chemical structure (α, β unsaturated carbonyls) were found to inhibit the *in vitro* growth of *Clostridium perfringens*.^128^ Fingolimod and its homolog sphingosine were also found to be potent inhibitors of *C. perfringens*.^128^ Other effects of fingolimod on the gut microbiome included increases in atypical forms of *E. coli*, *Enterobacter*, *Proteus* and *Parvimonas micra*,^124^ as well as reductions in *Bacteroides finegoldii*, *Roseburia faecis* and *Blautia* species.^41^

There is little evidence of other DMTs’ impact on microbial composition. Treatment with natalizumab was associated with reductions in *Bacteroides uniformis*, *Prevotella* species and *Bifidobacterium longum* and an increase in *Phascolarctobacterium* sp., while decreases of *Bacteroides finegoldii* and *Blautia* sp. were observed in patients treated with anti-CD20 antibodies.^41^ The effects of alemtuzumab were observed only in the monkey model of EAE, with profound alterations in *Lactobacillales*, *Enterobacterales*, *Clostridiales*, *Prevotella* and *Faecalibacterium* abundance.^129^

Despite some diversity in the reported effects of DMT on particular bacterial taxa, their overall impact is associated with reshaping composition of the gut microbiota in favor of anti-inflammatory strains. It was suggested that DMTs exert their immunomodulatory/immunosuppressive effects within the intestinal environment. IFN-β and teriflunomide were observed to mediate local proliferation of Tregs. Fingolimod was demonstrated to regulate migration of lymphoid cells from intestinal lamina propria and regulate maturation of plasmablasts in Peyer’s patches. By blocking migration of T cells, natalizumab was suggested to reduce their exposure to microbial antigens and subsequent activation and expansion. Some of the DMTs (DMF, GA and alemtuzumab) have also been found to stabilize the intestinal barrier and promote tissue integrity.^15,123^

Other mechanisms explaining DMT effects on the gut microbiota seemed to include shared metabolic pathways. Pathways of bacterial metabolism of retinol and methane were identified as targets of DMF and GA.^123^ As fumarates are degraded in the citrate cycle, an increase in late citrate cycle intermediates was observed during treatment with DMF, both in the patients’ serum and in the gut microbes’ metabolism.^130^ The inhibitory effect of DMF on *Clostridium perfringens* growth was neutralized by glutathione (having anti-oxidative properties), which suggests modulation of oxidative stress as another potential link between DMT and bacteria.^128^ Findings from a large panel of metabolomic analysis in serum and stools of MS patients^41^ demonstrated that pathways related to synthesis of lysine, L-ornithine, sugar nucleotides and unsaturated fatty acids were specifically modulated by particular DMTs. The most remarkable alterations were induced by fingolimod and IFN-β. Depletion of *F. prausnitzii*, a bacterium producing SCFA, and microbial pathways leading to pyruvate production was suggested to account for the low level of pyruvate, acetate and propionate in feces and serum from MS patients receiving these therapies. Increased absorption of propionate derived from bacteria, via upregulation of the SCFA transporter MCT1, was also hypothesized as a contributing mechanism.^41^

An interesting thread of investigation focused on the relationships between alterations in gut microbiota and side effects of DMT. With regard to DMF, one of the pilot studies^27^ failed to link microbial composition with gastrointestinal complaints, common in the early phase of treatment. Interestingly, in another study,^130^ it was found that a particular baseline microbiome signature (presence of *A. muciniphila* and absence of *P. copri*) could predict occurrence of lymphopenia, a relevant side effect of DMF.

The findings from studies on DMT’s impact on the gut microbiome encouraged further investigation into the therapeutic interventions modifying microbial composition, which would complement treatment with DMT (optimizing response to treatment, compromising their side effects) or affect other disease outcomes, not addressed so far.

## Pharmacological interventions targeting the gut microbiome in MS

### Probiotics

Probiotics are live microorganisms which confer a health benefit on the host, when administered in adequate amounts. They can interact with the gut microbiome and have an impact on the immunological system and the CNS function (e.g. through neurotrophic factors and neurotransmitters activity).^131^ Thus investigation of the role of probiotic supplementation affecting the course of MS has been undertaken.

Studies on animal models demonstrated some positive effects of *Lactobacillus*, *Escherichia coli* and *Prevotella* strains (administered orally or intraperitoneally), which prevented development of EAE or ameliorated its course.^132^

Marmoset twins were divided into two groups and fed either a yogurt-based (YBD) or water-based (WBD) diet before being immunized. The twins on YBD had less demyelination and a reduced pro-inflammatory response from T cells, B cells and cytokines. Some of the marmosets on YBD did not display any symptoms of EAE. The composition of the gut microbiome was only altered in these marmosets after they were immunized, likely due to the interplay between their diet and immune system responses.^133^

Administration of a probiotic cocktail to mice during chronic phase of TMEV infection reduced severity of the disease and emerging motor disability, as well as affecting composition of the gut microbiota, with increased abundance of *Bacteroidetes, Actinobacteria*, *Tenericutes* and *TM7* taxa. Related findings included reduced gliosis, infiltration of leukocytes and expression of IL-1β and IL-6 in the CNS and increased levels of butyrate and acetate in plasma.^134^

Probiotics were reported to promote integrity of the intestinal barrier and have an immunomodulatory effect (increasing the level of regulatory and anti-inflammatory cell subsets and their products and suppressing pro-inflammatory ones).^135^ As discussed before, bacterial metabolites (e.g. SCFAs) might also contribute to the reduction of regional and systemic immune-mediated responses.^136^ However, some contradictory findings were also reported, with *Lactobacillus reuteri* strains contributing to exacerbation of EAE, probably as a result of interaction with other bacteria and activation of molecular mimicry.^132^

There is scarce evidence for beneficial effect of probiotics in pwMS. The results of a few clinical trials showed that administration of probiotics (e.g. capsules containing *Lactobacillus* and *Bifidobacterium* strains) was associated with improvement in measures of disability, depression and general health, as well as reduced expression of pro-inflammatory cytokines, and favorable effects on markers of insulin resistance, profile of lipids and NO metabolites.^137–139^

The study of Tankou et al.^140^ focused particularly on probiotics’ impact on the gut microbiome. Following administration of a probiotic mix (*Lactobacillus, Bifidobacterium* and *Streptococcus*) for two months, the composition of microbiota changed in MS patients and HS with an increase in the relative abundance of mentioned species and a decrease in α-diversity. Reduced expression of CD14 and CD80 on peripheral monocytes was also observed. Microbial and immunological alterations ceased after withdrawal of probiotics. Due to the relatively small sample size, short duration and a number of confounding factors (dietary habits, DMT used), the relevance of findings from these trials is limited and further studies seem to be necessary.^8^

### Antibiotics

Bactericidal or bacteriostatic effects of antibiotics are bound to influence the gut microbiome; therefore antibiotics have been considered as possibly beneficial interventions in MS.

In several studies on experimental models of CNS autoimmune inflammation, oral administration of broad spectrum antibiotics before immunization prevented or ameliorated the disease.^141,142^

Gut dysbiosis was found in a non-obese diabetic murine model of EAE, resembling SPMS. Diminished disease severity and mortality in mice treated with a mixture of broad-spectrum antibiotics suggested reciprocal effects between CNS inflammatory demyelination and composition of the gut microbiome.^45^

Modifications of gut microbiota in EAE mice (decreased phylogenetic diversity and richness, lowered *Firmicutes*/*Bacteroidetes* ratio) were associated with an increased Treg response^143^ and altered function of iNKT cells.^144^ Following antibiotic administration, suppression of molecules produced by *L. reuteri* and *Erysipelotrichaceae* was also observed and a subsequent decrease in accumulation of MOG specific Th17 cells.^145^ The oral administration of antibiotics to mice infected with TMEV induced profound but temporary dysbiosis and modified immune-mediated responses, with lower levels of CD4^+^ and CD8^+^T cells in the CNS and in peripheral lymph nodes. Moreover, increased mortality in the course of TMEV infection was observed after cessation of antibiotics.^57^

However, the impact of antibiotics was limited when they were used during already established autoimmune inflammation, and so was their effect on oligodendrocyte progenitor cell differentiation and remyelination.^146^ In contrast to oral treatment, intraperitoneal administration of ampicillin caused worsening of EAE; a concomitant decrease in bacteria able to transform tryptophan into AhR agonists indicated a potential pathomechanism.^101^

These findings were not translated into clinical investigations. There were inconsistent results from the studies analyzing the relationships between risk of MS and use of antibiotics due to infections.^147,148^ Two small trials showed a positive effect of doxycycline (combined with IFN-β) or minocycline on clinical and radiological indices of MS activity, but the composition of gut microbiota was not taken into account.^149,150^ Despite some promising results, adverse effects of long-term treatment with antibiotics (including growth of opportunistic and resistant pathogens) are a significant barrier to use of these interventions in pwMS.

## Dietary interventions

Despite established the substantial role of pharmacological treatment (mainly DMT) in management of MS, there is still ongoing interest in the supportive role of dietary interventions and their ability to influence MS-related outcomes. Particular components of diet or dietary regimens were investigated in view of their impact on the course of disease (frequency and severity of relapses), its particular symptoms (e.g. pain or fatigue), but also general health issues and quality of life. Due to diversity in design of the trials and investigated variables, consistent and conclusive evidence of the benefit from dietary intervention in pwMS is still lacking.^151^

**Figure 5.**
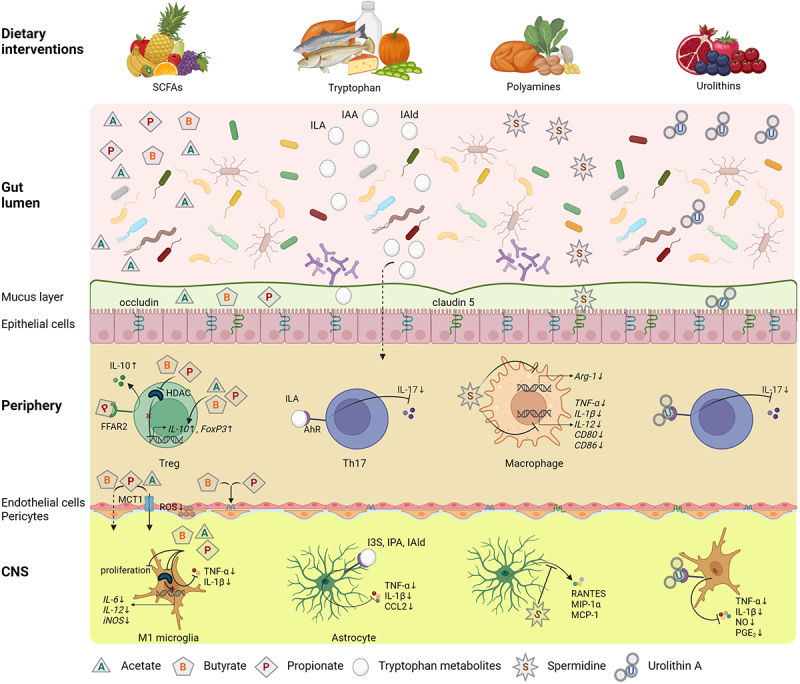
Effects of dietary intervention upon the gut dysbiosis and processes involved in MS pathology.

A significant thread of investigation of dietary interventions in MS is focused on their interaction with the gut microbiome through multiple pathways. Particular diet compounds, as metabolic substrates, may directly support the growth of some bacterial strains and inhibit others, thus shaping the microbial composition. Indirect effects include the impact of diet on host-microbiota immune interactions, e.g. the immune sampling within intestinal Peyer’s patches. Furthermore, diet may affect integrity and function of the intestinal barrier. Considering these links and potential to target the gut microbiome with various dietary interventions, their putative role in a complex therapeutic approach to MS has been investigated (Figure 5).^152,153^

SCFAs, tryptophan, polyamines and urolithin, derived from diet components, are the most relevant metabolites which can interact with immune and nervous system.

Gut bacteria are known to produce major SCFAs (acetate, propionate and butyrate), spermidine (the end product of L-arginine metabolism) and urolithins, as well as increase tryptophan metabolites (ILA, IAA, IAld).

SCFAs modulate Tregs in the systemic circulation by promoting transcription of genes encoding *IL-10* and *FoxP3*, by inhibition of HDAC, and stimulate production of IL-10 via FFAR2-dependent manner. ILA and urolithin A inhibit Th17 polarization and reduce production of IL-17 via AhR. Spermidine inhibits the expression of pro-inflammatory cytokines and co-stimulatory molecules in macrophages through downregulating the activity of NF-κB pathway, as well as upregulates expression of *Arg-1* in macrophages.

SCFAs cross the BBB to enter CNS by difusion or via MCT1. They can increase BBB integrity by restoring tight junction proteins (occludin and claudin 5) expression and by preventing ROS production in the endothelial cells.

In the CNS, SCFAs inhibit production of TNF-α, IL-1β, IL-6, IL-12 and iNOS by pro-inflammatory microglia M1 and limit their proliferation. Urolitin A decreases secretion of pro-inflammatory markers (TNF-α, IL-1β, NO, PGE_2_). Tryptophan metabolites (I3S, IPA and IAld) inhibit TNF-α, IL-6 and CCL2 production from astrocytes in an AhR dependent manner, whereas spermidine supresses astrocyte-derived chemokines (MIP-1α, MCP-1, RANTES).

Abbreviations: AhR – aryl hydrocarbon receptor, Arg-1 – arginase 1, CCL2 – chemokine (C-C motif) ligand 2, CNS – central nervous system, FFAR2 – free fatty acid receptors 2, FoxP3 – forkhead box P3, HDAC – histone deacetylase, I3S – indole-3-sulfate, IAA – indole-3-acetic acid, IAld – indole-3-aldehyde, IL – interleukin, ILA – indole-3-lactic acid, iNOS – inducible nitric oxide synthase, IPA – indole-3-propionic acid, MCP-1 – monocyte chemoattractant protein-1, MCT1 – proton-dependent monocarboxylate transporter 1, MIP-1α – macrophage inflammatory protein-1α, NO – nitric oxide, PGE_2_ – prostaglandin E_2_, RANTES – regulated upon activation, normal T cell expressed and secreted, ROS – reactive oxygen species, SCFAs – short chain fatty acids, Th – T helper, TNF-α – tumor necrosis factor alpha, Treg – regulatory T cell.

### Dietary components

#### Vitamin D

A low level of vitamin D is considered as a risk factor for MS development and activity, with moderate strength of evidence from randomized trials.^2,154^ Some beneficial effects of vitamin D on inflammatory markers and clinical and/or radiological measures of MS activity have been reported.^155,156^ However, the concomitant effects of DMT, as well as interactions between sun exposure, geographical distribution and dietary intake of vitamin D, must be considered.^2^

With regard to links with the gut microbiome, it is known that deficiency of vitamin D3 affects intestinal calcium absorption, disturbs gut homeostasis and increases intestinal permeability for bacteria and their products, which results in a concomitant rise in bacterial toxins.^14^ Investigation of the gut microbiome in HS with vitamin D deficiency showed only temporary changes in the *Firmicutes*/*Bacteroidetes* ratio during the restoration of vitamin level, while microbial α-diversity and β-diversity did not change throughout the study.^157^ Following vitamin D supplementation in overweight/obese healthy adults, higher abundance of *Lachnospira* and lower abundance of *Blautia* were observed.^158^ A study evaluating the effect of vitamin D supplementation on the bacterial community in MS female patients (untreated or receiving GA) showed an increase in *Faecalibacterium*, *Akkermansia* and *Coprococcus* genera in the untreated subgroup.^25^

#### Prebiotics

Prebiotics – the substrates used by intestinal bacteria – include fructans, galactooligosaccharides and so-called resistant starch. Indigestible for the host, they can be degraded by bacterial strains to products which feed other bacteria or enter further metabolic pathways, e.g. folates, indoles, secondary bile acids, trimethylamine-N-oxide (TMAO), lactate, succinate and SCFA. Administration of prebiotics may modulate the composition of the gut microbiome, supporting an increase in the number of *Bifidobacteria* and other SCFA producers. Furthermore, prebiotics can interact with the receptors of immune cells and induce cytokine expression.^159^

In an animal model, administration of dietary non-fermentable fiber, which prevents the development of EAE, was shown both to affect the composition of gut microbiota (overall decrease in diversity, with increased abundance of *Ruminococcaceae*, *Helicobacteraceae* and *Enterococcaceae* and reduced abundance of *Sutterellaceae*, *Lactobacillaceae* and *Coriobacteriaceae*) and to alter the metabolic profile, with an increase in long-chain fatty acids, reportedly promoting suppressive Th2 responses.^160^ In a clinical study, an association was detected between the dietary fiber intake in MS subjects and indices of systemic inflammation, anthropometric measures and disability level.^161^

#### SCFAs

As discussed earlier, a reduced level of SCFAs in MS subjects, possibly associated with alterations in the gut microbiome, might indicate beneficial effects of SCFA supplementation. Duscha et al.^63^ investigated this concept in a large cohort of MS subjects (including different disease types and DMT used) and noted initial depletion of *Butyricimonas* (SCFA producers) and enrichment of *Flavonifractor*, *Escherichia/Shigella* and *Collinsella* (promoted by low-SCFA conditions), accompanied by reduced amounts of propionic acid in the serum and stool of the patients (especially in treatment-naïve ones). Oral supplementation with propionic acid, believed to restore gut eubiosis, was found to cause induction of Tregs’ suppressive function and decrease Th1/Th17. Furthermore, it improved clinical measures of the disease, including the relapse rate and progression of disability.

Animal model studies showed that administration of SCFAs, specifically acetate, ameliorated the severity of EAE in an IL-10-dependent fashion.^88^ Similar findings indicated that colonization of microbiota derived from MS patients resulted in upregulated gene expression related with Tregs, but only when it was preceded by treatment with propionate.^62^ Furthermore, reduced CNS inflammation and demyelination were observed after preventive treatment with butyrate^162^ and propionate.^62^ Intriguingly, butyrate administration after disease onset had little impact on disease course,^162^ while propionate treatment in the same fashion resulted in recovery of axonal density, even though in the preventive approach butyrate reduced demyelination and immune cell infiltration.^62^ Thus immunomodulatory properties of SCFAs and their link with the gut microbiome may indicate a therapeutic potential for MS.

#### PUFAs

Polyunsaturated fatty acids (particularly omega-3 PUFA) are contained in plant or marine derived food products but also widely available as supplements of diet.

Studies on experimental models of MS have indicated a beneficial effect of PUFA exerted by their immunomodulatory features, inhibiting peripheral and CNS T cells’ activity. They were shown to prevent demyelination^163^ as well as promote neuroprotection and remyelination,^164^ which corresponded with amelioration of the disease and delay in its onset.^165^ Conjugated linolenic acid (CLA) supplementation caused an increase in myeloid-derived suppressor cells in the intestinal lamina propria, which was associated with suppression of inflammation. Principal component analysis and relative abundance of types of the gut microbiome revealed profound differences, e.g. increases in *Porphyromonadaceae*, *Lachnospiraceae*, *Bacteroides*, *Lactobacillus* and *Akkermansia* in affected animals. However, antibiotic treatment did not abrogate beneficial effects of CLA on the course of EAE, which challenges the concept of the gut microbiome mediating this impact.^165^

Clinical studies concerning effects of PUFA on MS outcomes have not been as conclusive as those on animal models and have produced conflicting results.^166,167^ The study by Fleck et al. showed that CLA intake in RRMS patients on DMT significantly improved the anti-inflammatory profiles and functional signatures of circulating myeloid cells. However, the composition of the human gut microbiome was not analyzed.^165^

#### Polyphenols

Polyphenols (together with other plant-derived compounds) may affect the composition of gut microbiota. The studies on HS revealed that polyphenols promote growth of *Clostridia*, *Bifidobacteria* and *Lactobacilli* and reduce the abundance of pathogenic strains of *Clostridium* and *Bacteroides*.^168^

So far, the effects of polyphenols have been examined in the experimental model of EAE, with partly contradictory results. Flavonoids have been reported to lower levels of pro-inflammatory cytokines and to alleviate symptoms of the disease,^169–171^ but also to delay recovery from its acute phase.^172^ Resveratrol was associated with exacerbation of EAE,^173^ yet supported remyelination in another preclinical MS model.^174^ There is scarce evidence from clinical studies of beneficial effects of polyphenols in MS. Treatment with nanocurcumin was reported to decrease the expression of pro-inflammatory cytokines and Th17 related parameters, increase Treg activity and restore the expression profile of miRNA, as well as to improve the level of disability and quality of life in RRMS subjects.^175^ However, the direct impact of polyphenols on the gut microbiome and their emerging therapeutic potential in MS need further investigations.

#### Sodium chloride

Increased NaCl intake, typical for the “western diet” (WD), affects local and systemic tissue inflammation and may alter the composition and function of the intestinal microbiome. Research has shown that a high concentration of NaCl in the extracellular environment leads to increased amounts of *Lachnospiraceae*, *Ruminococcus* and *Prevotella* spp. as well as decreased numbers of *Lactobacillus* in the gut of EAE mice.^176,177^ It has also been demonstrated that an increased NaCl level results in dysregulation of immune homeostasis, an example of which is preferred activation of pro-inflammatory M1 macrophages and Th17 cells and suppressed induction of anti-inflammatory M2 macrophages and Tregs, specifically in the gut lamina propria.^178^ The proposed scenario in this setting is as follows: depletion of *Lactobacillus* impedes the integrity of the intestinal barrier, prevents production of anti-inflammatory butyrate and coincides with increased Th17 and decreased Treg activity, most likely mediated by decreased production of indole-3-lactic acid, a tryptophan metabolite.^179^ These processes ultimately lead to exacerbation of EAE, which can be reversed or ameliorated following administration of *Lactobacillus*.^98^

Clinical studies on NaCl relevance for MS are quite limited and their results have been inconsistent. Farez et al. reported that a diet enriched in NaCl was related to increased numbers of demyelinating lesions in MRI and a higher relapse rate.^180^ However, other reports did not reveal an effect of high NaCl intake on the risk of MS development or time to subsequent relapse.^181–183^ These studies did not investigate the impact of NaCl dietary intake in pwMS on gut dysbiosis.

### Dietary regimens

#### Intermittent fasting and ketogenic diet

Chronic or intermittent restriction in food intake (with or without malnutrition) and a ketogenic diet, which mimics fasting conditions, are associated with the formation of ketone bodies, which are an alternative energy source. These conditions affect signaling pathways involved in inflammatory processes and oxidative stress (e.g. FoxO transcription factors, sirtuins, antioxidant enzymes) and stimulate expression of neurotrophic factors. Dietary restriction modifies the gut microbiome by inducing enrichment in anti-inflammatory strains and decreases in pro-inflammatory bacterial products. The impact of these dietary interventions on the CNS is further mediated by activation of the endocrine and autonomic function.^184,185^

The beneficial effects of fasting and a ketogenic diet have been demonstrated in several studies on a murine EAE model.^186–188^ These dietary interventions delayed the disease onset and attenuated or even reversed its motor and cognitive symptoms. Clinical improvement was accompanied by increased levels of corticosterone, adiponectin and immunoregulatory markers, while pro-inflammatory cytokines and cell subsets, as well as the production of reactive oxygen species, were suppressed. Furthermore, pathologic findings included a decrease in inflammatory infiltration and demyelination in the spinal cord, with the promotion of remyelination and regeneration of oligodendrocyte precursors.

In the study by Cignarella et al.,^189^ fasting was found to result in enrichment of the *Bacteroidaceae*, *Prevotellaceae* and *Lactobacillaceae* families in the murine intestine, with enhanced microbial antioxidant metabolic pathways. Interestingly, FMT from mice on intermittent fasting improved the course of EAE in the mice on a normal diet, which highlights the role of the gut microbiome in the impact of fasting on the CNS autoimmune condition.

Although the effects of various fasting regimens on outcomes of MS have been investigated, few of these studies considered their link with gut microbiota.

Cignarella et al., based on their findings from an animal model, undertook a pilot clinical trial with pwMS during a relapse, who were treated with corticosteroids and underwent intermittent fasting versus an optional diet. The abundance of *Faecalibacterium*, *Lachnospiracea incertae sedis* and *Blautia* in gut microbiota showed an increasing trend after intermittent fasting, and the level of *Faecalibacterium* correlated with a concomitant decrease in leptin.^189^

Swidsinski et al. analyzed the composition of the gut microbiome in MS patients during 6 months of a ketogenic diet. The total concentrations and diversity of bacterial groups were reduced at baseline. During the dietary intervention, these indices further decreased, but they returned to baseline values after 12 weeks and exceeded them significantly by weeks 23–24, approaching the values in HS. These fluctuations were especially relevant for *Akkermansia* strains.^22^

An ongoing clinical trial investigating clinical, immunological and metabolic outcomes of MS patients using fasting or a ketogenic diet is expected to provide further evidence of the links between the gut microbiome and these dietary interventions in MS.^190^

#### Other types of diet

In a longitudinal study of untreated RRMS subjects, Cantoni et al.^191^ analyzed relationships between the gut microbiome, immune and metabolic parameters, diet and clinical outcomes of the disease. Although alterations in the gut microbiome and corresponding immune dysregulation were found in MS subjects in comparison with HS, they were not affected by the overall composition of dietary intake. However, a specific link was identified between the consumption of meat with abundance of meat-associated blood metabolites and a decreased amount of *Bacteroides thetaiotaomicron* (a polysaccharide-digesting bacterium), as well as an increase in Th17 cells.

Saresella et al. analyzed gut microbiota composition and immunological profiles in two groups of MS patients, on a high-vegetable/low-protein (HV/LP) diet or on WD. The results showed that *Lachnospiraceae* family members were significantly more abundant while the number of IL-17 producing T cells was lower in the HV/LP group compared to the WD group. The HV/LP diet also impacted clinical measures: relapse rate and level of disability.^192^

Among other diet regimens considered as potentially beneficial in MS, the modified Paleolithic,^193^ elimination Wahls/Swank^194^ and low-fat plant-based diet^195^ have been tested in randomized clinical trials. The results, though inconsistent, showed some improvement in measures of manual dexterity, fatigue and quality of life. However, no effects of dietary interventions on the gut microbiome were evaluated in these studies.

## Faecal microbiota transplantation

FMT is an infusion of crafted stool samples from a healthy donor into the gastrointestinal tract of the recipient, in order to restore microbial balance (eubiosis). FMT has primarily been used in the treatment of *Clostridium difficile* infection as well as other inflammatory or functional disorders of the gastrointestinal tract.^196,197^ Furthermore, potential FMT effects have also been investigated in systemic autoimmune diseases and disorders with primary or secondary CNS involvement.^198,199^

Regarding experimental studies on MS, FMT from healthy to immunized mice resulted in altered composition of their gut bacterial taxa and was associated with milder severity of the EAE symptoms, reduced expression of markers of neurodegeneration and diminished pathological symptoms (demyelination and axonal loss).^200^ An interesting study by Berer et al. conducted colonization of SJL mice (an animal model of spontaneous RRMS) with gut microbiota from human twins discordant for diagnosis of MS.^31^ Worsening of disease was observed only after application of stools derived from MS-affected individuals, accompanied by abundance of *Sutterella* strains and an enhanced immunoregulatory profile.

Investigation of the therapeutic potential of FMT in MS was encouraged by case reports, describing MS patients who were treated with this method due to severe gastrointestinal problems.^201^ Following FMT, they experienced a long-term (up to several years) stabilization of the disease course, with minimal improvement in disability and functional scores. A more recent single-subject study^202^, based on a year-long follow-up of an MS patient after double FMT, provided evidence for possible mechanisms of the beneficial effect of FMT. Improvement in measures of gait was accompanied by altered microbial parameters (abundance of *Faecalibacterium prausnitzii*, increased ratios of *Firmicutes*-to-*Bacteroidetes* and *Prevotellaceae*-to-*Bacteroidaceae*), increased concentrations of acetate, propionate and butyrate in successive stool samples, and enhancement of SCFA genomic pathways, which correlated with the serum level of brain derived neurotrophic factor.

A pilot randomized controlled trial was performed on a small group of RRMS patients, who received monthly FMTs as an early or late intervention for up to 6 months.^203^ Donor-specific alterations in gut microbiome composition were found after FMT, although high intraindividual variability prevented significance of modifications in α- and β-diversity of species. Elevated intestinal permeability in some of the subjects was normalized following a series of FMT. No significant changes in serum levels of pro-inflammatory and immunoregulatory biomarkers were observed throughout the study. However, the trial was prematurely stopped for non-medical reasons, so not all the planned outcomes were achieved.

Further studies (including ongoing phase 1 clinical trials) are expected to provide more data about the effects, tolerance and optimal procedure of FMT, which would further support implementation of this method in MS treatment.

## Other interventions

According to some reports, parasite infections could ameliorate clinical and radiological activity in MS. This effect (reversed by anti-helminth drugs) was attributed to induction of Tregs and secretion of immunosuppressive cytokines.^204,205^ Attempts have been made to evaluate the impact of intentional human hookworm infection on gut dysbiosis in MS, considering possible interactions between these microorganisms. Jenkins et al.^206^ observed significantly increased α-diversity in fecal samples of infected pwMS compared to the placebo group and differences in the abundances of a few bacterial taxa (e.g. *Parabacteroides*) which probably possess immunomodulatory functions. In the placebo group, greater abundance of the *Lachnospiraceae* family was found, considered to be associated with a shift toward a pro-inflammatory microbial profile and emerging disease progression. This observation seems contradictory to the previous findings because *Lachnospiraceae* members are SCFA producers.

Another interesting attempt to target the gut microbiome in pwMS was associated with physical activity. Barone et al.^207^ evaluated the influence of a multidimensional rehabilitation programme on various MS outcomes. The intervention resulted in improved gait efficiency and reduced fatigue, accompanied by a decrease in pro-inflammatory immune markers (CD4^+^/IFN-γ^+^Th1, CD4^+^/ROR-γ^+^ and CD4^+^/IL-17^+^ Th17) and serum concentration of lipopolysaccharide. In addition, significant changes were observed in the composition of gut microbiota. At baseline, pwMS presented with depletion in several bacterial families (*Lachnospiraceae* and *Ruminococcaceae*) and enrichment in others (*Coriobacteriaceae*, *Veillonellaceae*, *Prevotellaceae*, *Enterobacteriaceae*). Concerning their functions, there was a decrease in bacteria producing SCFA (*Roseburia*, *Coprococcus* and *Blautia*) and an increase in those exhibiting pro-inflammatory activity (*Collinsella* and *Prevotella*). After the rehabilitation programme, specific alterations of the gut microbiome included relative abundance of *Actinobacteria* (similar to HS), sustained predomination of *Bacteroidetes* and *Proteobacteria* over *Firmicutes*, as well as lowered levels (below reference values for HS) of *Ruminococcus* and *Dorea* (SCFA producers, pro-inflammatory profile). Furthermore, the decrease of *Collinsella* positively correlated with CD4^+^/ROR-γ^+^ Th17 levels, suggesting a link between this bacterial genus and the pro-inflammatory component of the autoimmune response.

## Conclusions

Altered composition and function of the gut microbiota, demonstrated in pwMS, are suggested to play a significant role in the complex background of the disease. Experimental and clinical studies have revealed the links between gut dysbiosis and a dysregulated immune response (mainly predominance of its pro-inflammatory components over regulatory ones) as well as bacterial metabolic pathways. Analysis of these host-bacteria interactions may provide a better insight into the processes contributing to MS development and affecting its course. Furthermore, investigation of gut dysbiosis in MS may enable clarification of currently known therapeutic targets and hopefully reveal potential new ones. Besides DMT, other interventions, targeted at gut microbiota, apparently have some potential to affect the activity and the course of the disease (Table 7). Due to problems with the translation of experimental findings into clinical models, further investigation and randomized trials are necessary to verify the beneficial effects of these interventions in MS.

**Table 7.** Experimental and clinical studies on gut commensal-based therapies and dietary interventions affecting gut microbiome in MS.

Intervention	Research model	Impact on the gut microbiome	Other effects	Reference
Probiotics	Experimental: rhMOG-induced EAE Marmoset twin pairs Yogurt-based (YBS) vs. water-based supplement	YBS – fed twins after immunization: *Bifidobacteria***↑**, *Bacteroides barnesiae***↓**, *Blautia stercoris***↓**, *M. formatexigens***↓**	Frequency of EAE induction from 100% to 75%**↓**, demyelination of the spinal cord **↓**, expression of Callitrichine herpesvirus 3**↓**	^133^
	Experimental: TMEV-IDD SJL/J mice Vivomixx-probiotic cocktail	*Bacteroidetes***↑**, *Actinobacteria***↑**, *Tenericutes***↑**, *TM7***↑**	Improvement in motor disability, microgliosis**↓**, astrogliosis**↓**, leukocyte infiltration**↓**, IL-10 gene expression**↑**, plasma levels of butyrate and acetate**↑**	^134^
	Clinical: 9 pwMS/13 HS Probiotic – VSL3	*Lactobacillus***↑**, *Bifidobacterium***↑**, *Streptococcus***↑**, *Akkermansia***↓**, *Blautia***↓**	Frequency of inflammatory monocytes**↓**, expression of CD80 on monocytes**↓**, expression of HLA on dendritic cells**↓**, alterations of microbial metabolic pathways associated with e. g. methane metabolism	^140^
Prebiotics	Experimental: Spontaneous EAE – OSE and wild-type C57BL/6 mice Non-fermentable fiber (cellulose) rich diet vs. control diet	Significantly altered Beta-diversity Decreased OTU richness in Alpha-diversity analysis *Ruminococcaceae***↑**, *Helicobacteraceae***↑**, *Enterococcaceae***↑**, *Sutterellaceae***↓**, *Lactobacillaceae***↓**, *Coriobacteriaceae***↓**	IFN-γ-producing Th1 cells in intestinal lamina propria**↓**, levels of IL-4 and IL-5 produced by Th2 cells**↑**, butyric acid**↓**, LCFAs**↑**	^160^
SCFAs	Experimental: MOG_35–55_-induced EAE C57BL/6 mice Propionic acid (PA) intake vs. lauric acid (LA) intake vs control diet	EAE did not change microbiome composition LA intake: shift toward *Prevotellaceae***↓** and *Bacteroidete*s**↓**	LA intake: more severe course of the disease, Th17 cells in the CNS**↑**, Th1 and Th17 cells in the spleen**↑**, LCFAs**↑**, SCFAs (particularly PA) in feces**↓** PA intake: restoring the altered balance between Tregs and effector T cells	^62^
PUFAs	Experimental: Spontaneous EAE -OSE IgH^MOG^xTCR^MOG^ mice Conjugated linoleic acid (CLA) intake vs. control diet	Profound changes in the overall composition of the microbiome (principal component analysis) *Porphyromondaceae***↑**, *Lachnospiraceae***↑**, *Bacteroides***↑**, *Lactobacillus***↑**, *Akkermansia***↑**	Ameliorated disease course, infiltration of immune cells in the spinal cord and the intestinal lamina propria**↓**, MDSC-like cells in the intestinal lamina propria**↑**, pro-inflammatory T cell responses**↓**	^165^
NaCl	Experimental: MOG_35–55_-induced EAE FVB/N mice High salt diet (HSD) vs normal salt diet	Lack of obvious pattern in overall microbial composition (based on OTUs) HSD: *Lactobacillus***↓**, *Oscillibacter***↓**, *Pseudoflavonifractor***↓**, *Clostridium XIVa***↑**, *Johnsonella***↑**, *Rothia Parasutterella***↑**	Altered level of fecal central carbon and nitrogen metabolites	^98^
Vitamin D	Clinical: 7 pwMS (5 GA-treated and 2 non-treated) vs. HS (both with vitamin D deficiency) Vitamin D3 5000 IU/d	After vitamin D supplementation: *Faecalibacterium***↑**, *Enterobacteriaceae***↑**, *Ruminococcus***↓** Non-treated pwMS: *Akkermansia***↑**, *Faecalibacterium***↑**, *Coprococcus***↑** GA-treated pwMS: *Janthinobacterium***↑** *Eubacterium***↓**, *Ruminococcus***↓**		^25^
Intermittent fasting	Experimental: Induced EAE model, -C57BL/6 mice Fasting every other day vs. normal diet	Overall increased gut bacteria richness, *Lactobacillaceae***↑**, *Bacteroidaceae***↑**, *Prevotellaceae***↑**	Th17 cells**↓**, Tregs**↑**, inflammatory cells infiltration and demyelination in spinal cord**↓**, antioxidative microbial metabolic pathways**↑**, synthesis and degradation of ketone bodies**↑**, glutathione metabolism**↑**, lipopolysaccharide biosynthesis**↓**, leptin**↓**, adiponectin**↑**	^189^
	Clinical: 16 pwMS IER vs. free caloric intake	*Faecalibacterium***↑**, *Lachnospiracea incertae sedis***↑**, *Blautia***↑** *Faecalibacterium* abundance correlated with adiponectin level	Plasma B cells**↓**	^189^
Ketogenic diet	Clinical: 25 pwMS (ketogenic vs. normal diet), 14 HS	*Roseburia***↓**, *Bacteroides***↓**, *Faecalibacterium prausnitzii***↓**		^22^
Other dietary regimens	Clinical: 24 untreated pwMS and 25 controls Balanced diet (intake of basic types of food)	*Faecalibacterium***↓**, *Prevotella***↓**, *Lachnospiracea*e**↓**, *Anaerostipes***↓** (OTU relative abundance) Correlation between microbiota and level of disability *B. thetaiotaomicron***↓** correlating with consumption of meat	Th17cells**↑**, abundance of meat-associated blood metabolites – associated with consumption of meat**↑**	^191^
	Clinical: 20 pwMS High-vegetable/low-protein diet (HV/LP) vs. “Western Diet”	HV/LP subgroup: *Lachnospiraceae***↑**	IL −17 producing T cells**↓**, relapse rate**↓**, disability level**↓**	^192^
FMT	Experimental: MOG_35–55_-induced EAE C57BL/6 mice FMT-treated vs. non-treated immunized mice vs. normal controls	Increased Shannon index for α-diversity after FMT FMT-treated EAE mice: *Bacteroidetes***↑** *Firmicutes***↓**, *Tenericutes***↓**, *Cyanobacteria***↓** (shift toward the levels typical for normal controls) EAE severity scores: *Lachnoclostridium*, *Lachnospiraceae* - negative correlation, *Mollicutes_RF9, Ruminococcaceae, Turicibacter, Thalassospira -* positive correlation	Activation of microglia and astrocytes**↓**, improvement of BBB integrity	^200^
	Experimental: Germ-free RR SJL/J mice FMT from pairs of human twins discordant for MS	FMT from healthy twin: *Adlercreutzia***↑**, *Tannerella***↑**, *Ruminococcus***↓** FMT from MS twin: *Sutterella***↓** FMT from healthy twin: *Adlercreutzia***↑**, *Tannerella***↑**, *Ruminococcus*↓	IL-10 production**↓**	^31^
	Clinical: Single MS patient, 2 FMT 12-months of follow-up	Increased microbial richness *Firmicutes-to-Bacteroidetes***↑**, *Prevotellaceae-to-Bacteroidaceae***↑**, *Faecalibacterium prausnitzii***↑**	Concentration of acetate, propionate and butyrate in feces**↑**, SCFA genomic pathways**↑**, serum level of BDNF**↑**, improved measures of gait	^202^
	Clinical: 9 pwMS Monthly FMTs for up to 6 months	At baseline: *Bacteroides***↑**, *Blautia faecis***↑**, *Bacteroides uniformis***↑**, *Prevotella***↓**, *Paraprevotella***↓** No significant change in microbiota diversity following repeated FMTs	Partial normalization of elevated intestinal permeability, pro- or anti-inflammatory markers unchanged	^203^

The alterations are indicated by arrows. Abbreviations: BDNF – brain derived neurotrophic factor, EAE – experimental autoimmune encephalomyelitis, FMT – fecal microbiota transplantation, GA – glatiramer acetate, HLA – human leukocyte antigen, IER – intermittent energy restriction, OSE – spontaneous opticospinal encephalomyelitis, OTU – operational taxonomic unit, PUFAs – polyunsaturated fatty acids, pwMS – people with multiple sclerosis, (rh) MOG – recombinant human myelin, oligodendrocyte glycoprotein, SCFAs – short chain fatty acids, TMEV-IDD – Theiler’s murine encephalomyelitis virus (TMEV)-induced demyelinating disease.

## Data Availability

Data sharing is not applicable to this article as no new data were created or analyzed in this study.

## Abbreviations

AhR: aryl hydrocarbon receptor
Akt: serine/threonine protein kinase
AMPK: adenosine monophosphate-activated protein kinase
APC: antigen presenting cell
Arg-1: arginase 1
BACE1: β-site amyloid precursor protein cleaving enzyme 1
BBB: blood-brain barrier
BDNF: brain derived neurotrophic factor
CCL2: chemokine (C-C motif) ligand 2
CD: Crohn’s disease
CNS: central nervous system
cPLA_2_: cytosolic phospholipase A_2_
CSF: cerebrospinal fluid
DC: dendritic cell
DMF: dimethyl fumarate
DMT: disease modifying therapy
EAE: e*x*perimental autoimmune encephalomyelitis
EDSS: expanded disability status scale
ERK: extracellular signal-regulated kinase
FFAR: free fatty acid receptor
FMT: fecal microbiota transplantation
FoxP3: forkhead box P3
FTY720: fingolimod
GA: glatiramer acetate
GF: germ-free
GPR: G protein coupled receptor
HDAC: histone deacetylase
HLA: human leukocyte antigen
HS: healthy subjects
I3C: indole-3-carbinol
I3S: indole-3-sulfate
IAA: indole-3-acetic acid
IAld: indole-3-aldehyde
IDO: 1-indolamine 2,3-dioxygenase
IER: intermittent energy restriction
IL: interleukin
ILA: indole-3-lactic acid
iNKT: invariant natural killer T cells
iNOS: induced nitric oxide synthase
IPA: indole-3-propionic acid
JNK: Jun N-terminal kinase
LCFAs: long chain fatty acids
LFA-1: lymphocyte function associated antigen 1
MAPK: mitogen-activated protein kinase
MBP: myelin basic protein
MCP-1: monocyte chemoattractant protein-1
MCT1: proton-dependent monocarboxylate transporter 1
MHC-II: major histocompatibility complex class II
MIP-1α: macrophage inflammatory protein-1α
MOG: myelin oligodendrocyte protein
MS: multiple sclerosis
mTOR: mammalian target of rapamycin
NFE2L2: nuclear factor erythroid‐derived 2‐like 2
NFκB: nuclear factor kappa light chain enhancer of activated B cells
NMOSD: neuromyelitis optica spectrum disorder
NO: nitric oxide
Nrf2: nuclear factor erythroid 2-related factor 2
NZ: natulizumab
ODC-1: ornithine decarboxylase 1
Orai1: calcium release-activated calcium modulator 1
OSE: spontaneous opticospinal encephalomyelitis
OUT: operational taxonomic unit
PGE_2_: prostaglandin E_2_
PI3K: phosphoinositide 3-kinase
PPMS: primary progressive multiple sclerosis
PUFAs: polyunsaturated fatty acids
pwMS: people with multiple sclerosis
PXR: pregnane X receptor
RA: rheumatoid arthritis
RANTES: regulated upon activation, normal T cell expressed and secreted
(Rh) MOG: recombinant human myelin, oligodendrocyte glycoprotein
ROS: reactive oxygen species
RRMS: relapsing-remitting multiple sclerosis
S1P: sphingosine 1-phosphate
SAT-1: spermidine/spermine N1 acetyltransferase 1
SCFAs: short chain fatty acids
SMCT1: sodium-coupled monocarboxylate transporter 1
SPMS: secondary progressive multiple sclerosis
STIM: stromal cell interaction molecule
Th: T helper
TLR: toll-like receptor
TMEV-IDD: Theiler’s murine encephalomyelitis virus (TMEV)-induced demyelinating disease
TNF-α: tumor necrosis factor alpha
Treg: regulatory T cell
UC: ulcerative colitis
VDR: Vitamin D Receptor
VEGF-B: vascular endothelial growth factor B
WD: western diet
